# 4E-BP1 acts as a molecular rheostat balancing regenerative healing and fibrotic scarring

**DOI:** 10.1038/s12276-026-01724-0

**Published:** 2026-06-02

**Authors:** Hanyu Dou, Jianzhou Li, Lin Lin, Mengyu Jin, Jingyuan Wang, Hequn Fu, Jiongming Lu, Qinyi Chen, Leihong Xiang, Juan Wang, Xiaolei Ding

**Affiliations:** 1 Institute of Geriatrics, Affiliated Nantong Hospital of Shanghai University (The Sixth People’s Hospital of Nantong), School of Medicine, Shanghai University, Nantong, China; 2 Shanghai Engineering Research Center of Organ Repair, School of Medicine, Shanghai University, Shanghai, China; 3 College of Life Sciences, University of Chinese Academy of Sciences, Beijing, China; 4 Shanghai Institute of Nutrition and Health, University of Chinese Academy of Sciences, Chinese Academy of Sciences, Shanghai, China; 5 Department of Dermatology, Huashan Hospital, Fudan University, Shanghai, China

**Keywords:** Molecular biology, Cell biology

## Abstract

Efficient wound healing relies on tightly coordinated protein synthesis to support the complex cellular activities underlying tissue repair. However, the mechanisms governing translational control during tissue regeneration remain incompletely defined. Here, we identify the mTORC1 effector 4E-binding protein 1 (4E-BP1) as a critical regulator of wound repair and fibrotic remodeling. Phosphorylated 4E-BP1 was markedly increased in wounds and fibrotic tissues, indicating dynamic engagement of the mTORC1/4E-BP1 signaling axis during repair. Functional studies revealed that genetic ablation of 4E-BP1, mimicking fully phosphorylated 4E-BP1, enhanced re-epithelialization, angiogenesis, and granulation tissue formation in wound tissues, yet concurrently promoted myofibroblast activation and excessive collagen deposition, and fibrotic progression in bleomycin-induced skin fibrosis. Conversely, sustained overexpression of 4E-BP1 impaired wound closure and attenuated fibrotic responses. Moreover, in vitro, 4E-BP1 expression directly governed transforming growth factor-β1-mediated fibroblast collagen synthesis. Phosphorylated 4E-BP1 levels and related transcriptional signatures were elevated in human skin fibrotic scar tissues. These findings demonstrate that 4E-BP1 acts as an mTORC1-downstream effector that shapes the balance between reparative efficiency and fibrotic remodeling. Targeting the mTORC1/4E-BP1 signaling axis may therefore offer novel therapeutic opportunities to optimize wound healing and prevent pathological scarring.

## 4E-BP1 regulates wound repair and fibrosis balance

Skin serves as a vital barrier environmental barrier with remarkable regenerative capacity. This study explores the role of mTORC1 and its downstream effector 4E-binding protein 1 (BP1) in skin wound repair and fibrosis. Using genetic mouse models, the authors demonstrate that the absence of 4E-BP1 accelerates wound closure, enhances angiogenesis, and increases collagen deposition, whereas its overexpression delays healing. 4E-BP1 regulates protein synthesis, influencing cell proliferation and ECM formation. These findings highlight 4E-BP1 as a crucial factor in balancing effective tissue repair and fibrotic progression. Modulating 4E-BP1 activity may improve wound repair while minimizing excessive scarring, offering a potential therapeutic strategy for managing chronic wounds and fibrotic skin disorders. Future studies could focus on developing pharmacological agents targeting 4E-BP1 to optimize wound healing outcomes.

This summary was initially drafted using artificial intelligence, then revised and fact-checked by the author.

## Introduction

Skin provides a crucial barrier against environmental insults and possesses a remarkable regenerative capacity. Upon injury, wound healing proceeds through tightly regulated phases: hemostasis, inflammation, proliferation, and remodeling, which coordinately restore tissue integrity and barrier function^1,2^. This complex process involves clot formation, inflammation, granulation tissue formation, and re-epithelialization, ultimately culminating in scar formation^1–3^. The quality of healing is influenced by factors including age, metabolic status, and disease burden. When disrupted, these processes can lead to chronic wounds, malignant transformation, or pathological fibrosis^4–6^.

During tissue repair, both resident and recruited cells undergo extensive metabolic rewiring, characterized by enhanced nutrient uptake and utilization, particularly amino acids, glucose, and lipids, which governs regenerative outcomes^7–9^. This metabolic restructuring provides cellular fuel necessary for energy-intensive regenerative processes, including extensive extracellular matrix (ECM) synthesis, rapid cell proliferation, and coordinated tissue remodeling. Dysregulation of the signaling networks that orchestrate the balance between catabolic breakdown and anabolic reconstruction can severely compromise healing efficiency or precipitate pathological disorders, including the development of chronic non-healing wounds and pathological scars^2,10^. Despite these critical roles, the molecular mechanisms that integrate nutrient sensing with translational control during skin repair and fibrosis remain incompletely characterized^11^. Specifically, how translational machinery components regulate mRNA translation to direct wound healing outcomes have been underexplored.

The mechanistic target of rapamycin (mTOR) is a highly conserved serine/threonine kinase that functions as a master regulator of cellular growth, metabolism, and tissue homeostasis^12,13^. mTOR signaling operates through two structurally and functionally distinct complexes, mTORC1 and mTORC2, with mTORC1 acting as a central integrator of nutrient and growth factor cues to drive anabolic metabolism^14,15^. Previous work from our group and others has demonstrated that precise regulation of mTOR activity is essential for skin morphogenesis^16^. Genetic disruption of mTORC1 impairs epidermal barrier formation^17,18^ and delays wound healing, as observed across multiple species, including flies and mice^19–21^. Clinically, the application of mTORC1 inhibitor rapamycin and its analog leads to disturbed healing responses, emphasizing the contribution of the pathway in tissue repair^22^. Conversely, hyperactivation of mTORC1, such as through loss of upstream negative mediators, such as PTEN or TSC, accelerates wound closure but often promotes tumorigenesis, fibrotic overgrowth, and excessive scarring^19,23,24^. Together, these findings highlight the need for a finely tuned mTORC1 signaling threshold to support optimal wound healing while preventing pathological fibrosis.

A principal effector pathway downstream of mTORC1 governs protein synthesis through the phosphorylation of ribosomal proteins and the eukaryotic translation initiation factor 4E-binding proteins (4E-BPs)^12,25^. Among these, 4E-BP1 serves as a key regulator of protein translation initiation via binding to eIF4E, preventing the assembly of the translational initiation complex and thereby repressing translation^25,26^. Upon mTORC1 activation, 4E-BP1 is phosphorylated and releases eIF4E, thereby licensing selective mRNA translation. Notably, reduced mTOR signaling and the consequent low 4E-BP1 phosphorylation have been observed in diabetic wounds and is associated with impaired healing capacity^27–29^. Furthermore, emerging evidence implicates the 4E-BP1/eIF4E axis in regulating fibrotic processes^30–32^. Inhibition of mTORC1/4E-BP1 axis attenuates transforming growth factor-β1 (TGF-β1)-induced fibrotic response in human tenon, lung, and kidney fibroblasts in vitro^30–33^. Despite these advances, the in vivo function of mTORC1-mediated translational control via 4E-BP1 in skin repair and fibrosis remains insufficiently defined.

In this study, we investigated the functional role of 4E-BP1 in skin wound healing and fibrotic remodeling by using genetic loss-of-function and gain-of-function approaches in mouse models. We demonstrate that genetic ablation of 4E-BP1, which mimics sustained mTORC1-dependent translational activation, accelerates wound closure, promotes angiogenesis, and enhances collagen deposition. By contrast, sustained overexpression of 4E-BP1 impairs re-epithelialization and delays tissue repair. Transcriptomic analysis further revealed that gene sets regulated by the 4E-BP1/eIF4E axis are enriched in human scar tissues, supporting its clinical relevance. These findings position 4E-BP1 as a critical mTORC1-downstream effector that governs the balance between reparative efficiency and fibrotic remodeling. Our data underscore the therapeutic potential of modulating mTORC1/4E-BP1 signaling as a strategy to enhance wound healing outcomes while minimizing excessive scarring.

## Materials and methods

### Animal

All animal experiments were approved by the Institutional Animal Care and Use Committee of Shanghai University (ECSHU 2022-100). The mice were housed in a standard specific pathogen-free condition. *Eif4ebp1*^−^/^−^ mice were obtained from Shanghai Model Organisms. The body weight of mice was monitored weekly from weeks 3 to 8 after birth. The experiments were conducted with littermate controls. Genotyping was conducted through PCR analysis of genomic DNA extracted from tail tips. The sequences of primers used were as follows: P1, 5ʹ-TTC TGC CAC CGT CAT CCC TA-3′; P2, 5′-AGC TAC CGA ACC CCT CGA AT-3′; P3, 5′-AAT CGG AGA GTT CTG CCA CC-3′; P4, 5′-GGA TCC CGA CGT ATC CTC CA-3′.

### AAV-mediated 4E-BP1 overexpression

The recombinant adeno-associated virus (AAV) vector overexpressing *Eif4ebp1* (pAAV[Exp]-CMV > mEif4ebp1) and the corresponding control AAV-EGFP vector were purchased from VectorBuilder.

For in vitro experiments, cells were infected with AAV-*Eif4ebp1* or control virus at a multiplicity of infection of 1 × 10^5^ and incubated for 96 h before subsequent analyses. For in vivo experiments, viral stocks were diluted 1:5 in PBS before use, resulting in a working concentration >4 × 10^12^ GC/ml. Six-week-old wild-type mice received subcutaneous injections of either 100 μl of diluted virus or control virus at four dorsal sites (25 μl per site). Skin samples were collected 3 weeks after injection to confirm 4E-BP1 overexpression.

### Wounding experiments

Excisional wounds were created and harvested in accordance with established protocols^34^. Both male and female mice between 6 and 9 weeks of age were used. After removal of hair, four full-thickness-excisional skin wounds with a 6-mm diameter were created on the back of each mouse using biopsy punches. Afterwards, the mice were housed individually. The wounds were photographed at 0, 4, 7, and 14 day post-injury (dpi) and subsequently collected for further analysis.

### Bleomycin-induced skin fibrosis

Skin fibrosis model was established as established protocols^35^. Bleomycin (Cat. No. S1214, Selleck) was dissolved in sterile NaCl at a concentration of 1 mg/ml. Eight-week-old male mice were anesthetized and received daily subcutaneous injections of 100 μl bleomycin into a defined area of shaved dorsal skin. Injections were administered once daily for 5 consecutive days followed by a 2-day break with a cycle repeated four times. Control mice received an equal volume of NaCl. The fibrotic skin tissues were harvested at 14 and 28 days post-injection for subsequent analyses.

### Human skin samples

Human keloid skin samples were obtained from patients undergoing surgical excision at Huashan Hospital, Fudan University, with approval from the Ethics Committee of Huashan Hospital (HIRB Approval No. 2024-027). All procedures were conducted in accordance with the principles of the Declaration of Helsinki. Collected tissues were fixed, paraffin-embedded, and sectioned into 4-μm-thick slices, which were mounted on adhesive glass slides and subsequently processed for immunofluorescence staining.

### Histological analysis

The skin tissues were fixed with 4% paraformaldehyde solution, embedded in paraffin, and sectioned at a thickness of 4 μm. Subsequently, the sections were stained with hematoxylin and eosin (H&E) according to established protocols. Images were captured using a light microscope (Keyence, BZ-X810).

Immunohistochemistry (IHC) staining was performed as reported previously^36^. The sections were deparaffinized in xylene, rehydrated through graded ethanol series, and subjected to antigen retrieval in citrate buffer (pH 6.0). Endogenous peroxidase activity was blocked with 3% hydrogen peroxide, and non-specific binding sites were blocked with 5% bovine serum albumin. Subsequently, sections were incubated with primary antibodies including vascular endothelial growth factor (VEGF)-A (Cat. No. ab51745, Abcam) and α-smooth muscle actin (α-SMA) (Cat. No. AA132, Beyotime) at 4 °C overnight. The slides were then incubated with horseradish peroxidase-conjugated secondary antibodies. Visualization was performed using diaminobenzidine substrate, and sections were counterstained with hematoxylin, dehydrated, and mounted before microscopic examination.

For immunofluorescence staining, following fixing and blocking, the sections were incubated with the following primary antibodies at 4 °C overnight: p-4E-BP1 Thr 37/46 (Cat. No. 2855, Cell Signaling Technology), p-S6 Ser240/244 (Cat. No. 5364, Cell Signaling Technology), COL1A1 (Cat. No. 72026, Cell Signaling Technology), Ki67 (Cat. No. ab15580, Abcam), and CD31 (Cat. No. 557355, Becton, Dickinson and Company). Next, the sections were incubated with fluorescently labeled secondary antibodies: Goat Anti-Rabbit IgG (H + L) Highly Cross-Adsorbed Secondary Antibody, Alexa Fluor™ 594 (Cat. No. A-11037, Thermo Fisher Scientific) and Goat Anti-Rabbit IgG (H + L) Highly Cross-Adsorbed Secondary Antibody, Alexa Fluor™ 488 (Cat. No. A-11008, Thermo Fisher Scientific), at room temperature for 1 h. Nuclei were counterstained with 4′,6-diamidino-2-phenylindole. Slides were imaged using a confocal fluorescence microscope.

### Cell culture

Primary fibroblasts were isolated from the dorsal skin of C57BL/6 mice by explant culture. Briefly, 0.5 cm^2^ skin fragments were placed dermis-side down in culture dishes and maintained in DMEM supplemented with 10% fetal bovine serum and 1% penicillin/streptomycin. Fibroblasts began to migrate out from dermal explants after 5–7 days, with cells expanded to subconfluence before serial passaging. Human dermal fibroblasts (HDFs) derived from the foreskin tissue were purchased from the Chinese Academy of Sciences (Cat. No. SCSP-106). Both cell types were cultured in a humidified incubator with 5% CO_2_ at 37 °C. Cells at fewer than five passages were used in this study.

### Quantitative real-time PCR (qRT-PCR)

The RNA was extracted with TRIzol reagent (Cat. No. 15596018CN, Invitrogen). Isolated RNA was reverse-transcribed using the HiScript III RT SuperMix for qPCR kit (Cat. No. R323, Vazyme). qRT-PCR was performed using SYBR Green kit (Cat. No. Q711, Vazym). The 2^−^^ΔΔ^Ct method was used to determine the relative expression level of the target genes, with normalization to *GAPDH*. The following primer sequences were used: mouse *Gapdh* forward (AGG TCG GTG TGA ACG GAT TTG), reverse (TGT AGA CCA TGT AGT TGA GGT CA), mouse *Vegfa* forward (TGT ACC TCC ACC ATG CCA AGT), reverse (CGC TGG TAG ACG TCC ATG AA), mouse *Col1a1* forward (AGC TTT GTG GAC CTC CGG CT), reverse (ACA CAG CCG TGC CAT TGT GG), human *GAPDH* forward (GGC AAA TTC CAT GGC ACC G), reverse (ATG ACG AAC ATG GGG GCA TC), human *COL1A1* forward (GAG GGC CAA GAC GAA GAC ATC), reverse (CAG ATC ACG TCA TCG CAC AAC).

### Western blot

Cells and tissues were lysed with radioimmunoprecipitation assay buffer (Cat. No. P0013K, Beyotime) containing protease inhibitors (Cat. No. ST506, Beyotime) and phosphatase inhibitors (Cat. No. G2007, Servicebio). Protein concentration was determined by bicinchoninic acid assay (Cat. No. P0010, Beyotime). About 20 μg of protein was loaded to tris-polyacrylamide gel for SDS–PAGE and transferred to polyvinylidene fluoride membranes. After blocking with 5% non-fat milk in TBST, the membranes were incubated with primary antibodies, followed by incubation with secondary antibodies. The following primary antibodies were used: Smad2-pS465/467 (Cat. No. 18338, Cell Signaling Technology), p-Smad3 Ser423/425 (Cat. No. 9520, Cell Signaling Technology), Smad2/3 (Cat. No. 5678, Cell Signaling Technology), p-4E-BP1 Thr37/46 (Cat. No. 2855, Cell Signaling Technology), 4E-BP1 (Cat. No. 9644, Cell Signaling Technology), p-S6 Ser240/244 (Cat. No. 5364, Cell Signaling Technology), S6 (Cat. No. 2317, Cell Signaling Technology), COL1A1 (Cat. No. 72026, Cell Signaling Technology), α-SMA (Cat. No. AA132, Beyotime), β-actin (Cat. No. 3700, Cell Signaling Technology), VEGF-A (Cat. No. ab51745, Abcam), and GAPDH (Cat. No. 10494-1-AP, ProteinTech).

### Transmission electron microscopy (TEM) and collagen fibril analysis

Briefly, 2 mm^2^ pieces of back skin were fixed in 2% glutaraldehyde/2% formaldehyde in 0.1 M cacodylate buffer (pH 7.3) for 48 h at 4 °C followed by incubation with 2% osmium tetroxide (OsO_4_) in 0.1 M cacodylate buffer for 2 h at 4 °C. Samples were dehydrated through an ascending ethanol series, transferred to propylene oxide, and embedded in epoxy resin. Semi-thin sections were prepared for orientation purposes. For collagen fibril analysis, cross-sectioned collagen fibrils of the lower dermis were imaged at 15,000× magnification. Collagen fibril quantification was partially automated. Fibril circularity was calculated according to ref. ^37^ using the formula 4*πA*/*p*^2^ = circularity, in which *p* is the perimeter and *A* is the transverse area of the fibril. This parameter reaches a maximum value of 1 in circular objects. Objects with a circularity value < 0.6 were identified as artifacts and excluded from all further calculations. The mean fibril diameter and its standard deviation (SD) were calculated for each animal to assess the intra-individual coefficient of variation (CV) by the formula: SD/Mean × 100 = CV.

### Single-cell RNA sequencing (scRNA-seq) analysis of human skin

scRNA-seq data of human normal skin, wound, and pathological scar tissues were downloaded from GSE241132 and GSE163973. For GSE241132, samples with designated conditions including normal skin, wound 1 dpi, and wound 7 dpi generated by ref. ^38^ were used in this study. For GSE163973, scar tissue scRNA-seq data (GSM4994382, GSM4994383, and GSM4994384) generated by ref. ^39^ were used in this study.

### Data processing

ScRNA-seq data were processed using the Seurat R package (v5.0.1)^34^. Raw count matrices were imported and normalized, and batch effects were corrected using the CCA (canonical correlation analysis) algorithm^40^. Cell-type annotation was performed based on original cell type information derived from two previously published studies. To assess gene set activity across cells, we applied the AddModuleScore function in Seurat. Gene sets were curated from the Molecular Signatures Database (MSigDB, GSEA) and are detailed in Supplementary Table 1.

### Statistics

Statistical analysis was performed using GraphPad Prism 9 (GraphPad Software Inc.). Significance of difference was analyzed with two-tailed unpaired *t* test, one-way analysis of variance, and two-way analysis of variance. The data are presented as mean ± SD with a *P*-value less than 0.05 considered statistically significant. Results were presented as the mean derived from a minimum of three separate trials.

## Results

### Phosphorylated 4E-BP1 accumulates in wound scar and fibrotic tissues

**Fig. 1:**
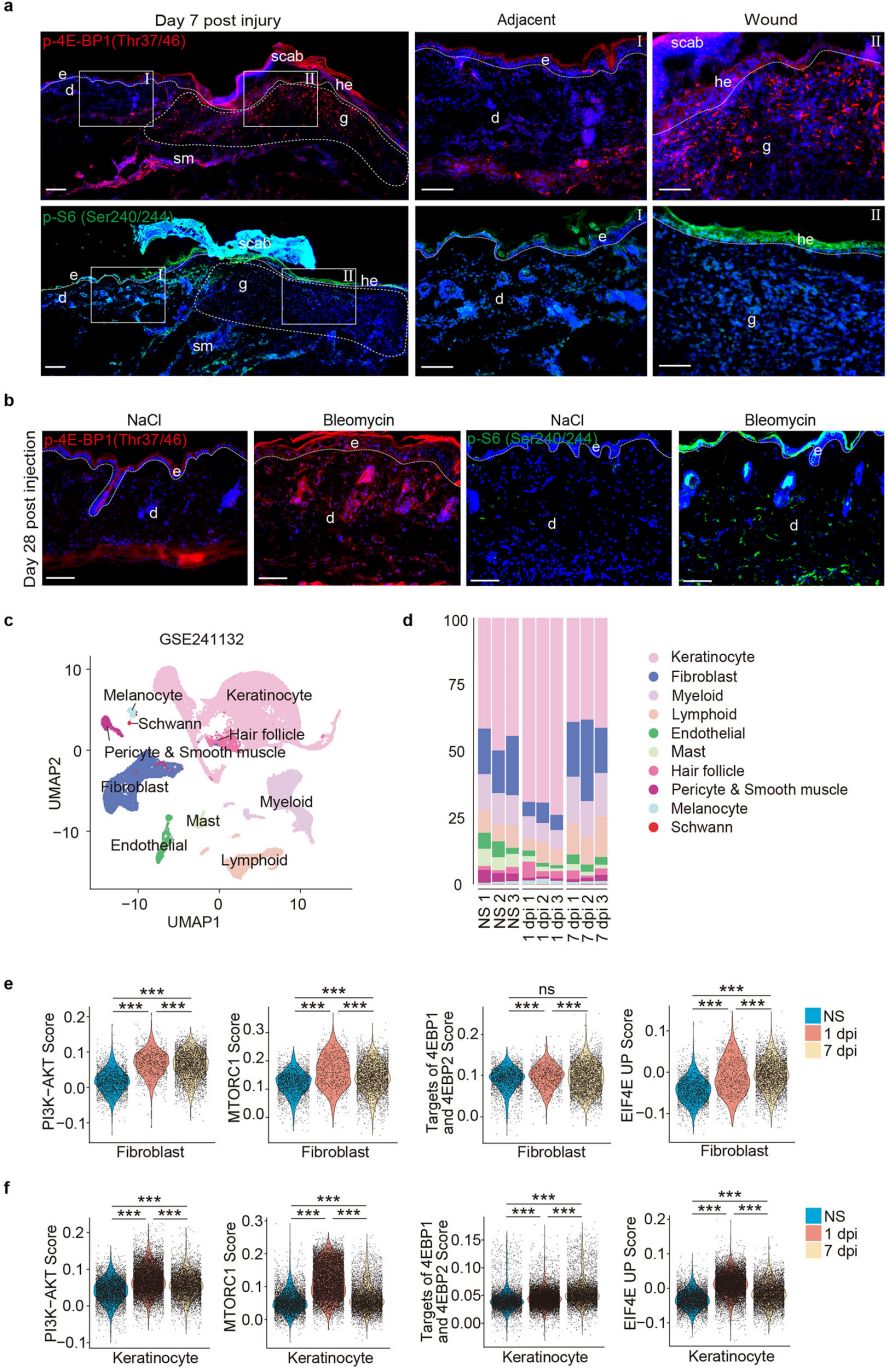
mTORC1/4E-BP1 signaling is activated in wound and fibrotic skin. **a** Representative immunofluorescence staining of phosphorylated 4E-BP1 (p-4E-BP1 Thr37/46) and phosphorylated S6 ribosomal protein (p-S6 Ser240/244) in full-thickness skin wounds at day 7 dpi. Left: low magnification view. scale bar, 200 μm. Right: high magnification of boxed region. Scale bar, 100 μm. **b** Immunostaining of p-4E-BP1 Thr37/46 and p-S6 Ser240/244 in skin sections from mice treated with bleomycin for 28 days to induce dermal fibrosis (scale bar, 100 μm). The dashed line indicates the junction between the epidermis and dermis. **c** Uniform manifold approximation and projection (UMAP) of all cell types, colored by annotated clusters. **d** Fraction of each major cell type out of all cells at indicated dpi. **e**, **f** Enrichment of PI3K–AKT–mTORC1 axis and 4E-BP1/eIF4E regulated gene signatures in skin lesions at indicated time points. BP binding protein, d dermis, dpi days post-injury, e epidermis, **g** granulation tissue, he hyperproliferative epithelium, mTOR mechanistic target of rapamycin, ns not significant, NS normal skin, sm skeletal muscle. *P < 0.05, **P < 0.01, ***P < 0.001 by Student’s unpaired two-tailed *t* test (parts **e** and **f**).

To characterize the activation status of the mTORC1/4E-BP1 signaling during wound repair and fibrotic remodeling, we performed immunofluorescence analysis targeting phosphorylated-4E-BP1 (p-4E-BP1) at Thr37/46, using phosphorylated S6 ribosomal protein (p-S6) at Ser240/244 as a canonical mTORC1 readout. In line with previous reports, p-S6 signals were markedly induced in wound epithelial cells at 7 dpi^19,41^. Concurrently, we observed pronounced accumulation of p-4E-BP1 in the basal layer of the migrating epithelial tongue, which was virtually absent from the epidermis of the intact skin. This elevated p-4E-BP1 expression extended throughout the underlying granulation tissue, indicating widespread activation of this signaling pathway during skin repair (Fig. 1a). Notably, p-4E-BP1 and p-S6 induction was already detectable at 1 dpi (Supplementary Fig. 1a), indicating the rapid activation of mTORC1 signaling following tissue damage. Consistently, western blot analysis of wound tissues further revealed strong activation of the mTOR signaling pathway (Supplementary Fig. 1b). TGF-β1 is a key cytokine to induce fibroblast differentiation into myofibroblasts. Similarly, treatment of primary mouse dermal fibroblasts with TGF-β1 also led to robust activation of mTOR signaling in vitro (Supplementary Fig. 1c). Importantly, we also detected strong p-4E-BP1 signal in a bleomycin-induced skin fibrosis experimental model. Challenges of mice with bleomycin in the skin strongly increased the levels of p-4E-BP1, when compared with non-fibrotic control (Fig. 1b). To assess the activation status of mTORC1/4E-BP1 signaling axis human wound healing, we analyzed publicly available scRNA-seq data sets of human normal skin and wound tissues^38^. Cells were categorized into 10 major cell types, including keratinocytes, fibroblasts, and immune cells, revealing dynamic shifts in cellular composition between homeostatic and injured skin (Fig. 1c,d). To probe pathway activity, we applied AddModuleScore to quantify enrichment of hallmark gene sets from the GSEA database, including PI3K–AKT, mTORC1, 4E-BP1/2 deficiency, and eIF4E overexpression signatures (Supplementary Table 1). Notably, both keratinocytes and fibroblasts from wounded skin displayed marked enrichment of PI3K–AKT–mTORC1 pathway gene signatures relative to normal counterparts (Fig. 1e,f). In particular, gene sets associated with loss of 4E-BP1and 2 function and eIF4E overexpression were significantly upregulated, consistent with a transcriptional signature of heightened cap-dependent translation (Fig. 1e,f). Collectively, these findings support the notion that persistent mTORC1/4E-BP1 activation is a shared feature of both physiological repair and pathological fibrotic remodeling, and it may serve as a key driver of tissue repair and fibrotic progression.

### 4E-BP1 loss accelerates wound healing

**Fig. 2:**
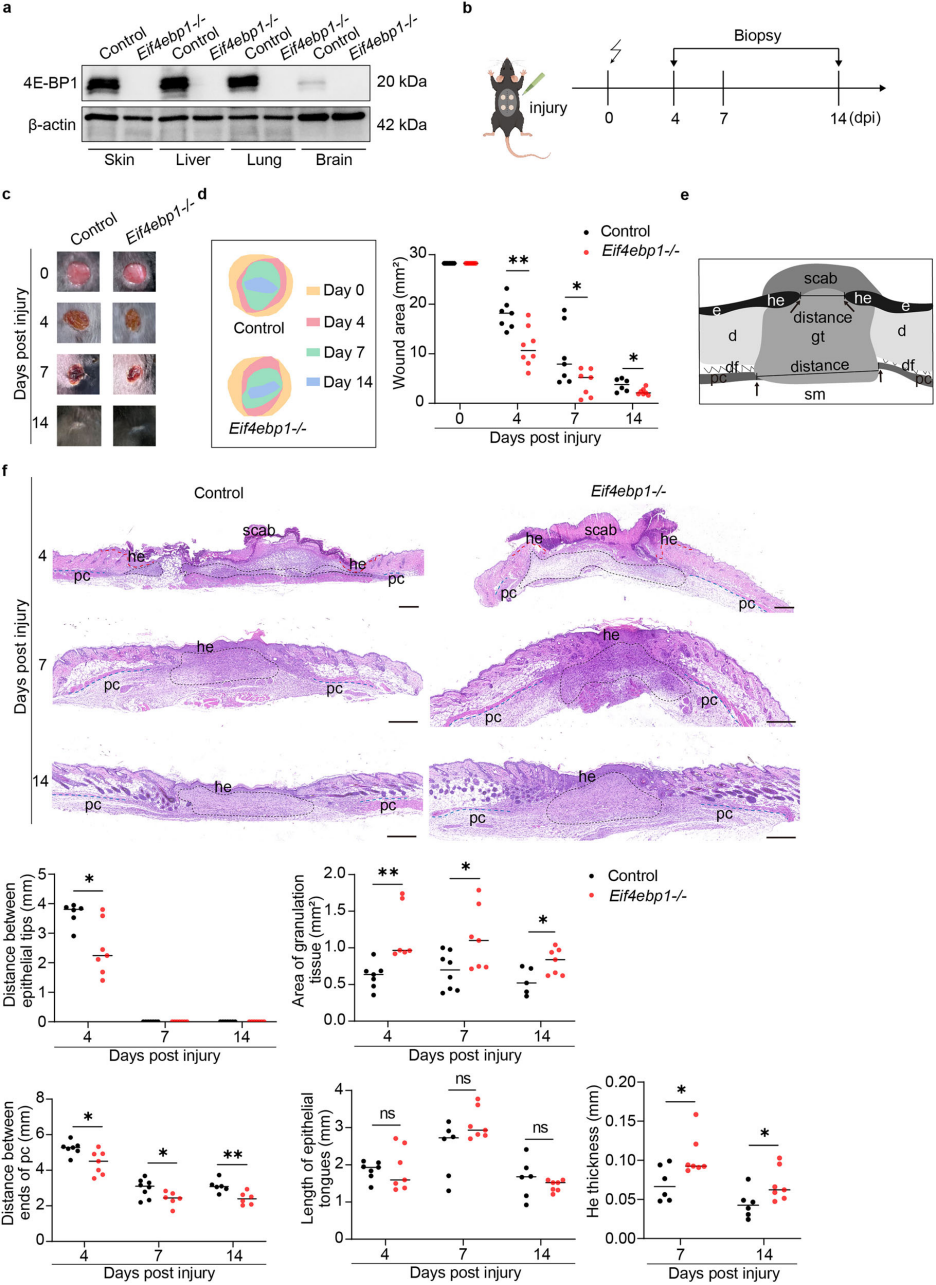
4E-BP1 loss accelerates wound healing. **a** Western blot analysis of 4E-BP1 protein expression in the skin, liver, lung, and brain of wild-type control and *Eif4ebp1*^−^/^−^ mice. **b** Schematic diagram illustrating the experimental design used for full-thickness-excisional wound healing assays. **c** Representative macroscopic images of wound closure in control and *Eif4ebp1*^−^/^−^ mice on days 0, 4, 7, and 14 dpi. **d** Wound closure traces over 14 days and quantification of wound area at the indicated time points (*n* = 7–8 wounds per group). **e** Scheme depicting the anatomical structure of skin wound. **f** Representative images of hematoxylin and eosin staining of wounded sections at 4, 7, and 14 dpi. Scale bar, 400 μm. Morphometric and quantitative analysis of wound closure kinetics. Each dot represents one wound (*n* = 6–8 wounds). BP binding protein, d dermis, dpi days post-injury, e epidermis, g granulation tissue, he hyperproliferative epithelium, pc panniculus carnosus, sfsubcutaneous fat tissue, sm skeletal muscle. *P < 0.05, **P < 0.01, ***P < 0.001 by Student’s unpaired two-tailed *t* test (parts **d** and **f**).

To investigate the functional role of mTORC1/4E-BP1 signaling in skin wound healing, we studied 4E-BP1-deficient mice, which mimic constitutive 4E-BP1 phosphorylation and thus sustained translational activation. Mice with targeted deletion of 4E-BP1 (*Eif4ebp1*^−^/^−^) were generated on a C57BL/6 background using CRISPR–Cas9-mediated genome editing (Supplementary Fig. 2a). Successful gene disruption was confirmed by PCR analysis of genomic DNA and immunoblotting, which demonstrated near-complete absence of 4E-BP1 protein across multiple tissues, including the skin (Fig. 2a and Supplementary Fig. 2b). In line with previous reports, *Eif4ebp1*^−^/^−^ mice were viable, fertile, and born at Mendelian ratios and exhibited no overt developmental abnormalities^42^. Histological examination of the skin revealed normal epidermal and dermal architecture. However, adult *Eif4ebp1*^−^/^−^ mice exhibited a modest but consistent reduction in body weight compared with wild-type littermates^42^ (Supplementary Fig. 2c–e).

To assess the functional consequences of 4E-BP1 loss on tissue repair, full-thickness-excision wounds were inflicted on the dorsal skin of *Eif4ebp1*^−^/^−^ and control mice, and healing dynamics was evaluated at defined time points post-injury (Fig. 2b). Macroscopic evaluation revealed significantly accelerated wound closure in *Eif4ebp1*^−^/^−^ mice. By day 4, wound area in mutants was reduced to ~40% of the initial size, compared with ~70% in control animals (Fig. 2c,d). Quantitative measurements further confirmed that wound areas remained consistently smaller in *Eif4ebp1*^−^/^−^ mice at both 7 and 14 dpi (Fig. 2d), indicating enhanced wound healing dynamics in the absence of 4E-BP1.

Morphological analysis using H&E staining of wound sections further corroborated the accelerated healing phenotype observed in *Eif4ebp1*^−^/^−^ mice. Compared with controls, wounds from *Eif4ebp1*^−^/^−^ mice exhibited a shorter distance between the epithelial tongue tips and also the ends of the panniculus carnosus and markedly increased granulation tissue. In addition, the thickness of hyperproliferative epidermis was significantly increased at 7 and 14 dpi (Fig. 2e,f). These morphological features indicate that 4E-BP1 loss accelerates epithelialization, granulation formation, and the enhanced contraction of the wound tissue, collectively contributing to the enhanced wound closure observed in *Eif4ebp1*^−^/^−^ mice.

**Fig. 3:**
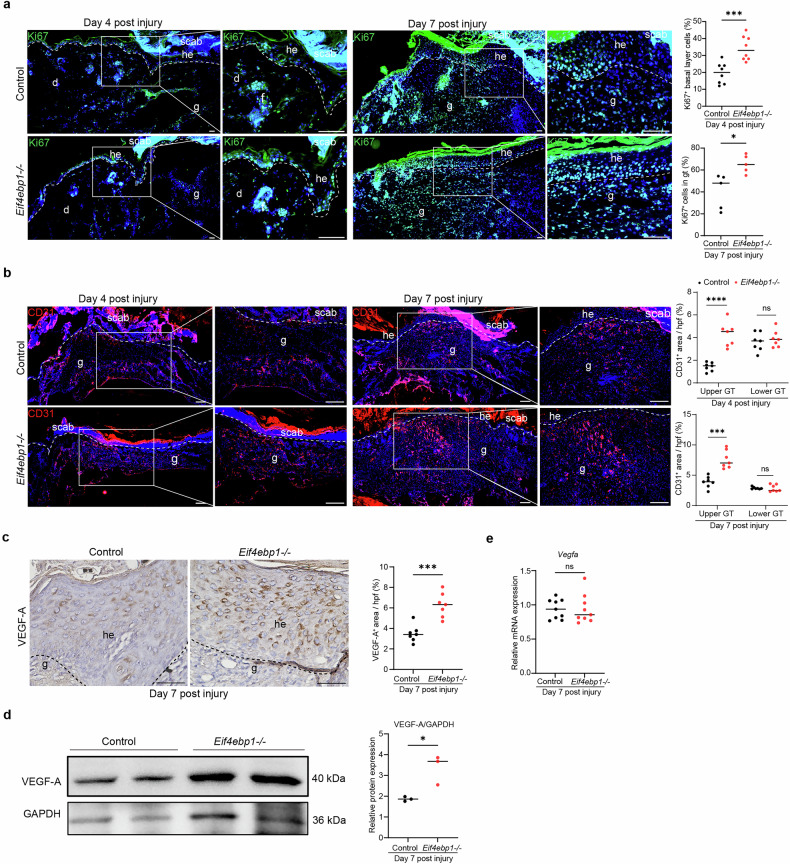
4E-BP1 loss enhances wound keratinocyte proliferation and angiogenesis. **a** Representative immunofluorescence staining of Ki67 on wound sections at 4 and 7 dpi. Scale bar, 50 μm. Quantification of the percentage of Ki67^+^ cells (*n* = 5–8 wounds). **b** Representative immunofluorescence staining of CD31 on wound sections at 4 and 7 dpi. Scale bar, 100 μm. Quantification of CD31⁺ area per hpf in superficial and deep granulation tissues (superficial GT and deep GT) of wound tissues. **c** Immunohistochemical staining of vascular endothelial growth factor (VEGF)-A on wounds at 7 dpi. Scale bar, 50 μm. Quantification of VEGF-A^+^ area per hpf (*n* = 6–7 wounds from five mice per genotype and time point). **d** Western blot analysis of VEGF-A protein from wounds at 7 dpi in control and *Eif4ebp1*^−^/^−^ mice (*n* = 3). Statistical results of western blot. **e** qRT-PCR analysis of *Vegfa* mRNA expression (*n* = 6–7 wounds from five mice per genotype). BP, binding protein; d, dermis; e, epidermis; g, granulation tissue; he, hyperproliferative epithelium; hpf, high-power field; ns, not significant. *P < 0.05, **P < 0.01, ***P < 0.001 by Student’s unpaired two-tailed *t* test (parts **a**–**e**).

Given the association between wound healing and cell proliferation, we evaluated the impact of 4E-BP1 deficiency on wound cell proliferation. Ki67 immunofluorescence staining was performed on wound tissues at 4 and 7 dpi. At 4 dpi, Ki67-positive cells were predominantly localized to the basal layer of the transitional epithelium near the wound edge. Quantitative analysis revealed a significant increase in the number of Ki67-positive epithelial cells in *Eif4ebp1*^−^/^−^ wounds compared with controls (Fig. 3a). By 7 dpi, increased Ki67-positive cells were also detected within the granulation tissue, suggesting that loss of 4E-BP1 enhances the proliferative capacity of both epithelial and stromal wound cell populations (Fig. 3a).

Angiogenesis is essential for wound healing ensuring adequate oxygen and nutrient delivery to support tissue regeneration^43^. To assess the impact of 4E-BP1 deficiency on wound angiogenesis, we performed CD31 immunofluorescence staining to visualize neovascular formation within granulation tissue. Robust neovascularization was observed in *Eif4ebp1*^−^/^−^ wounds, and morphometric analysis reveals significantly increased vascular density, in particular, the upper layers of granulation tissues compared with controls at both 4 and 7 dpi (Fig. 3b). VEGF-A, a key angiogenic mediator secreted by keratinocytes and other cell types during wound healing, is known to be translationally regulated by the 4E-BP1/eIF4E axis^44^. To determine whether the enhanced angiogenesis observed in *Eif4ebp1*^−^/^−^ mice was associated with altered VEGF-A expression, we performed IHC staining and western blot for VEGF-A on wound tissues at 7 dpi. VEGF-A protein levels were markedly elevated in *Eif4ebp1*^−^/^−^ wounds compared with controls (Fig. 3c,d). A similar increase was observed in human HaCaT keratinocytes following CRISPR–Cas9-mediated *EIF4EBP1* deletion (Supplementary Fig. 3). By contrast, qRT-PCR analysis revealed comparable *Vegfa* mRNA levels between the groups (Fig. 3e), suggesting post-transcriptional regulation. Collectively, these data indicate that mTORC1/4E-BP1 signaling promotes wound angiogenesis by enhancing epithelial-derived VEGF-A translation.

Given the translational regulation role of 4EBP1, we performed polysome profiling of wound tissues at 7 dpi to assess global protein synthesis rates and ribosomal activities. Polysome signals were increased in *Eif4ebp1*⁻/⁻ wounds compared to controls (Supplementary Fig. 4a), indicating the enhanced translational rates in the absence of 4E-BP1. We further performed proteomic analysis of the wound tissues to elucidate the regulatory mechanisms from a global perspective. Principal component analysis revelated an obvious separation between control and *Eif4ebp1*^−^/^−^ wounds, indicating substantial alterations in the wound proteome (Supplementary Fig. 4b). Differential expression analysis identified 149 upregulated and 119 downregulated proteins in the absence of 4E-BP1 (Supplementary Fig. 4c). Notably, several proteins associated with cell proliferation and fibrotic remolding, such as Rab5a and Smad2, were significantly upregulated in *Eif4ebp1*^−^/^−^ wounds (Supplementary Fig. 4d). Genes and Genomes (KEGG), Gene Ontology (GO), and protein interaction analysis demonstrated the enrichment of wound healing, cell apoptotic process, and fibronectin binding, known for promoting wound healing process (Supplementary Fig. 4e–g). These findings provide proteomic evidence that loss of 4E-BP1 facilitates a pro-regenerative and profibrotic translational program during skin repair.

### 4E-BP1 loss promotes myofibroblast activation and impacts collagen fibrillogenesis

**Fig. 4:**
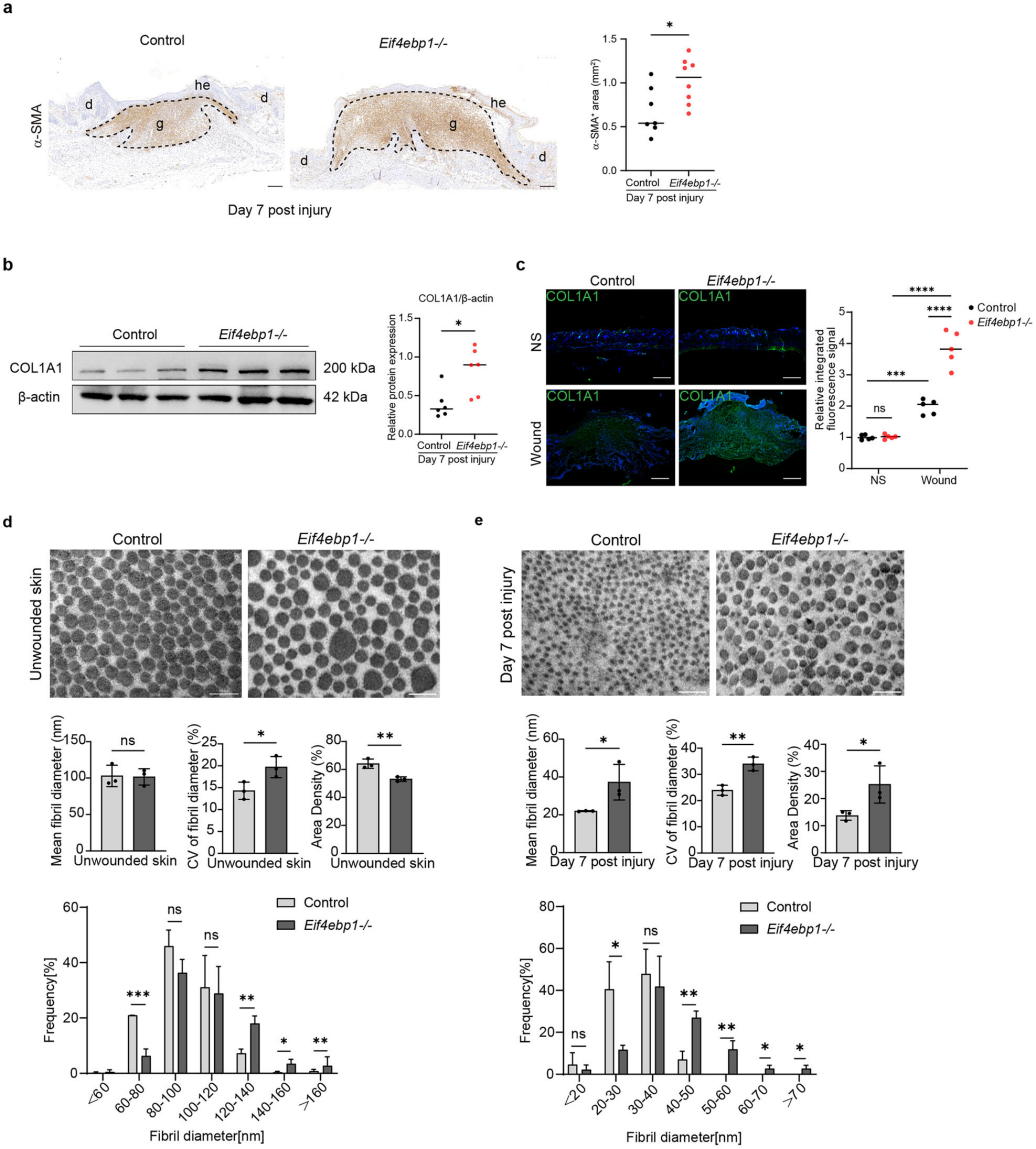
4E-BP1 regulates collagen synthesis during wound healing. **a** Representative immunohistochemical staining of α-smooth muscle actin (α-SMA) on wound sections at 7 dpi (*n* = 7–8 wounds from five mice per genotype). Scale bar, 200 μm. Quantification of α-SMA^+^ area. **b** Western blot analysis of COL1A1 protein from wounds at 7 dpi in control and *Eif4ebp1*^−^/^−^ mice. Statistical analysis of COL1A1 protein (*n* = 6 and non-parametric Mann–Whitney *U*-test was used). **c** Representative immunofluorescence staining of COL1A1 in normal skin and wounds at 7 dpi from control and *Eif4ebp1*^−^/^−^ mice. Quantification of COL1A1 fluorescence intensity (*n* = 5). Scale bar, 250 μm. **d** Ultrastructural analysis of collagen fibrils in unwounded skin (*n* = 3). Scale bar, 200 nm. Quantification of fibril index. **e** Ultrastructural analysis of collagen fibrils in wound granulation tissue at 7 dpi (*n* = 3). Scale bar, 200 nm. Quantification of fibril index. CV coefficient of variation, d dermis, g granulation tissue, he hyperproliferative epithelium, ns not significant, NS normal skin. *P < 0.05, **P < 0.01, ***P < 0.001 by Student’s unpaired two-tailed *t* test (parts **a**–**e**).

During wound healing, fibroblasts sense mechanical tension and differentiate into contractile α-SMA-expressing myofibroblasts, which are critical for ECM deposition and wound contraction^45^. Immunohistochemical analysis revealed a pronounced expansion of the α-SMA-positive area in the wounds from 4E-BP1-deficient mice (Fig. 4a), indicative of enhanced myofibroblast activation and accumulation. Myofibroblasts are major producers of ECM components such as collagen I (COL1A1) and fibronectin and are essential for matrix remodeling. Western blot and immunofluorescence staining analysis of skin tissues from 7 dpi demonstrated elevated COL1A1 deposition in *Eif4ebp1*^−^/^−^ wounds (Fig. 4b,c), suggesting that 4E-BP1 deficiency promotes matrix synthesis during the proliferative phase of repair.

To assess whether this enhanced collagen production was accompanied by changes in fibril ultrastructure, we performed TEM on both intact and wounded dermis. Compared with the control skin, collagen fibers of uninjured skin of 4E-BP1-deficient mice appeared less densely packed and more disorganized (Fig. 4d). At 7 dpi, TEM analysis of the healing dermis revealed a heterogeneous collagen network in mutant mice, with abnormally thin fibrils interspersed among hypertrophic fibers, whereas the control skin lacked such features and showed a more uniform fibrillar organization (Fig. 4e). Quantitative analysis confirmed an altered distribution of fibril diameters in *Eif4ebp1*^−^/^−^ mice, with increased mean fibril diameters and area density (Fig. 4e). These findings suggest that 4E-BP1 deficiency not only promotes myofibroblast-driven collagen deposition but also perturbs ECM organization, potentially impairing the mechanical stability and functional quality of the regenerated dermis.

### 4E-BP1 overexpression attenuates wound healing responses

**Fig. 5:**
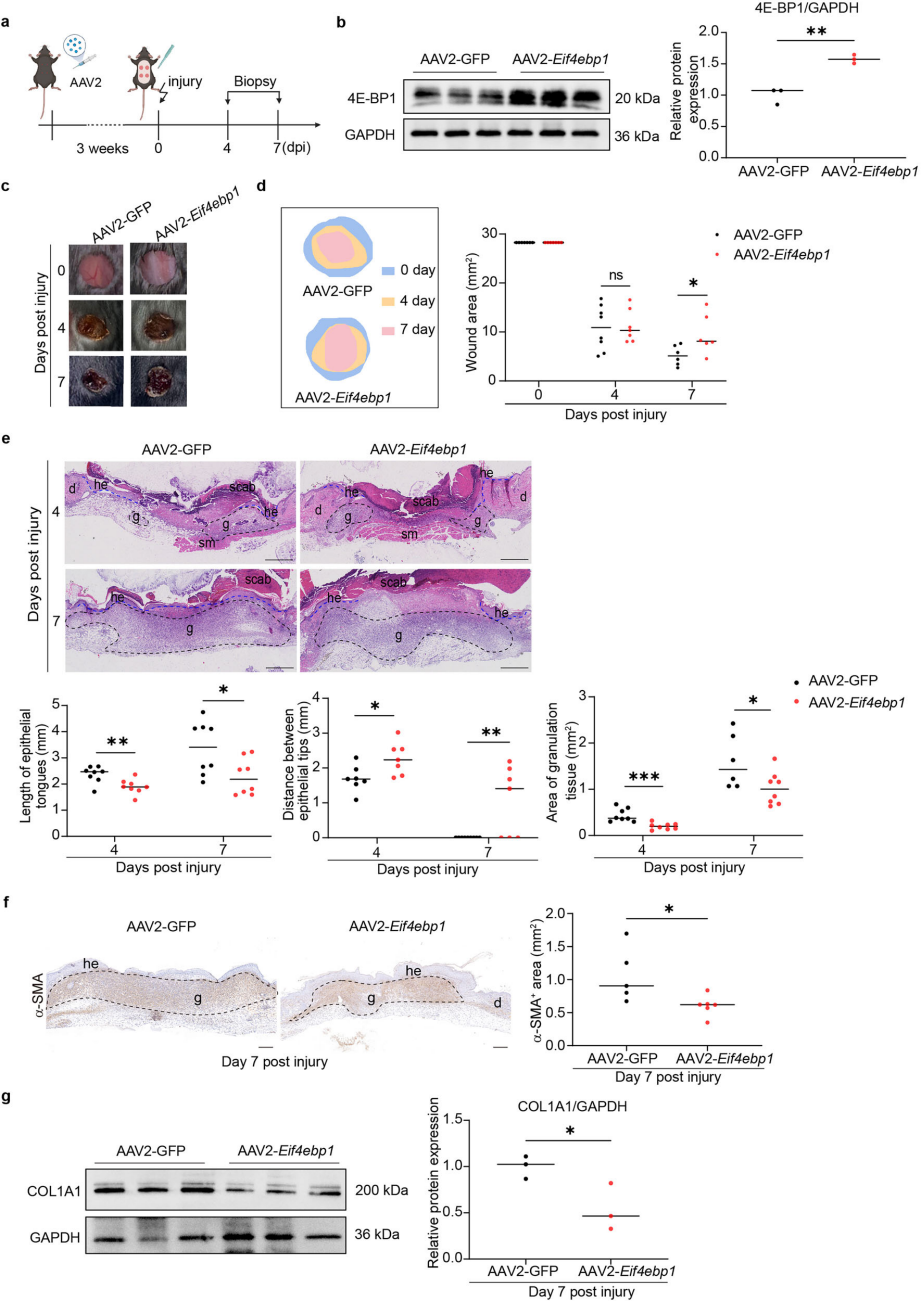
4E-BP1 overexpression impairs wound healing and suppresses collagen expression. **a** Schematic diagram illustrating experimental design. **b** Western blot analysis of 4E-BP1 in skin tissues isolated from control and 4E-BP1 overexpression mice (*n* = 3). Statistical analysis of 4E-BP1 expression levels. **c** Representative images of skin wounds in control and 4E-BP1 overexpression mice on 0, 4, and 7 dpi. **d** Wound closure traces over a 7-day period in both control and 4E-BP1 overexpression mice. The wound area was quantified on 0, 4, and 7 dpi (*n* = 6–8 per group). **e** Representative images of hematoxylin and eosin staining of wound sections at 4 and 7 dpi. Scale bar, 500 μm. Quantification of wound healing related parameters in control and 4E-BP1 overexpression mice. Each dot represents one wound (*n* = 6–8 wounds). **f** Representative immunohistochemical staining of α-smooth muscle actin (α-SMA) in wound sections at 7 dpi (*n* = 5–6 wounds). Scale bar, 200 μm. Quantification of α-SMA^+^ area. **g** Western blot analysis of COL1A1 protein in wound tissues at 7 dpi from control and 4E-BP1 overexpression mice (*n* = 3 wounds from three mice per genotype). Quantification of COL1A1 expression. GAPDH was used as loading control. AAV adeno-associated virus, BP binding protein, d dermis, dpi day post-injury, g granulation tissue, GFP green fluorescent protein, he hyperproliferative epithelium, ns not significant, sm skeletal muscle. *P < 0.05, **P < 0.01, ***P < 0.001 by Student’s unpaired two-tailed *t* test (parts **b**, **d** and **e**–**g**).

To further investigate the functional role of 4E-BP1 in skin repair, we generated mice with sustained 4E-BP1 overexpression via intradermal injection of an AAV vector (Fig. 5a and Supplementary Fig. 5a,b). Western blot analysis confirmed robust upregulation of 4E-BP1 protein levels in treated skin (Fig. 5b). Full-thickness excisional wounds were then introduced to assess wound healing dynamics (Fig. 5c,d). Macroscopic assessment of wound closure over time revealed no differences between groups at 4 dpi. However, by day 7, wound areas in 4E-BP1-overexpressing mice remained significantly larger than in AAV2-GFP treated mice (Fig. 5c,d), indicating a delay in wound closure.

Histological evaluation further supported this observation. H&E-stained sections revealed impaired re-epithelialization in 4E-BP1-overexpressing wounds, as evidenced by longer distances between epithelial tips and incomplete epidermal coverage at 7 dpi (Fig. 5e). The newly formed epithelium appeared more fragile, shorter, and thinner, indicating that sustained overexpression of 4E-BP1 imposed a supra-physiological, hyper-repressive translational state, constraining keratinocyte growth and migratory capacity, resulting in a measurable reduction in epithelial tongue length. Granulation tissue volume was consistently reduced across time points, pointing to compromised dermal regeneration. Wound contraction, assessed by measuring the distance between the ends of the panniculus carnosus, was unaffected (Fig. 5e), indicating that 4E-BP1 selectively impairs processes associated with epithelial and granulation tissue formation.

To examine the cellular basis of this delay, Ki67 immunofluorescence staining was performed and revealed a marked reduction in proliferating cells within the wound bed of 4E-BP1-overexpressing mice at multiple time points (Supplementary Fig. 5c). This suggests that excessive 4E-BP1 represses translation-dependent expansion of regenerative cell populations. Consistently, CD31 IHC showed a significant reduction in vascular density within the granulation tissue at both 4 and 7 dpi (Supplementary Fig. 5d), reflecting impaired angiogenesis. This defect correlated with diminished VEGF-A protein expression in the wound bed at day 7 (Supplementary Fig. 5e). α-SMA expression was significantly diminished in 4E-BP1-overexpressing wounds (Fig. 5f). Western blot analysis further demonstrated reduced COL1A1 levels in the wound bed (Fig. 5g). Together, these results indicate that increased 4E-BP1 expression functions as a negative regulator of key regenerative processes, including keratinocyte proliferation, angiogenesis, myofibroblast differentiation, and ECM production.

### 4E-BP1 deficiency promotes bleomycin-induced skin fibrosis

**Fig. 6:**
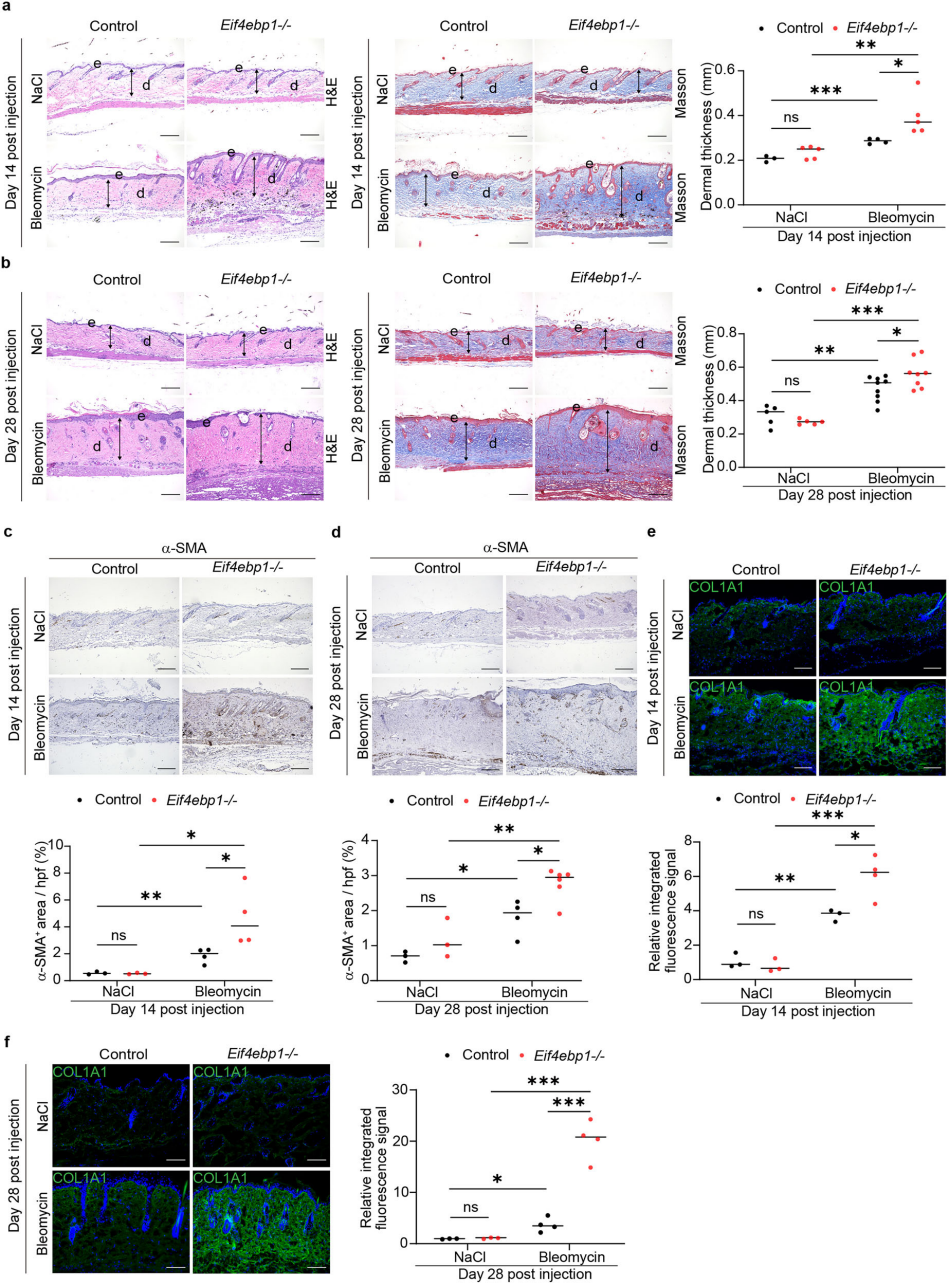
4E-BP1 deficiency exacerbates bleomycin-induced skin fibrosis. **a**, **b** Representative images of hematoxylin and eosin (H&E) and Masson’s trichrome staining of dorsal skin sections from control and *Eif4ebp1*^−^/^−^ mice at day 14 (part **a**) and day 28 (part **b**) post-injection of bleomycin or vehicle control. Quantification of dermal thickness (*n* = 3–8 skin samples). Scale bar, 250 μm. **c**, **d** Representative immunohistochemical staining of α-smooth muscle actin (α-SMA) in dorsal skin at day 14 (part **c**) and day 28 (part **d**) post-injection. Quantification of the percentage of α-SMA^+^ area (*n* = 3–6 skin samples). Scale bar, 250 μm. **e**, **f** Representative immunofluorescence staining of COL1A1 in dorsal skin at day 14 (part **e**) and day 28 (part **f**) post-injection. Quantification of COL1A1 fluorescence intensity (*n* = 3–4). Scale bar, 500 μm. d dermis, e epidermis, hpf high-power field, ns not significant. *P < 0.05, **P < 0.01, ***P < 0.001 by Student’s unpaired two-tailed *t* test (parts **a**–**f**).

To investigate whether loss of 4E-BP1 sensitizes skin to fibrotic remodeling beyond acute wound healing, we utilized the bleomycin-induced dermal fibrosis model^35^, which recapitulates key pathological features of human scleroderma (Supplementary Fig. 6a). Photographic images of dorsal skin and statistical analysis of body weight at 2 and 4 weeks post-bleomycin injection revealed more severe fibrotic skin lesions and pronounced weight loss in *Eif4ebp1*^−^/^−^ mice compared with controls (Supplementary Fig. 6b,c). Histological analysis of skin sections collected after 2 and 4 weeks of bleomycin administration revealed a pronounced fibrotic response in 4E-BP1-deficient mice compared with wild-type controls (Fig. 6a,b). Masson’s trichrome staining confirmed the excessive deposition of collagen-rich ECM, indicating enhanced fibrosis (Fig. 6a,b). Immunostaining analysis of α-SMA revealed a significant upregulation of α-SMA expression in 4E-BP1-deficient skin following bleomycin challenge (Fig. 6c,d). Similarly, COL1A1 expression levels were significantly elevated, indicating an increase in collagen synthesis (Fig. 6e,f). These findings demonstrate that 4E-BP1 acts as a molecular brake on fibroblast activation and ECM deposition during chronic fibrotic insult. In the absence of 4E-BP1, fibroblasts exhibit an exaggerated response to profibrotic cues, resulting in excessive matrix accumulation and dermal thickening.

### 4E-BP1 overexpression alleviates fibrosis

**Fig. 7:**
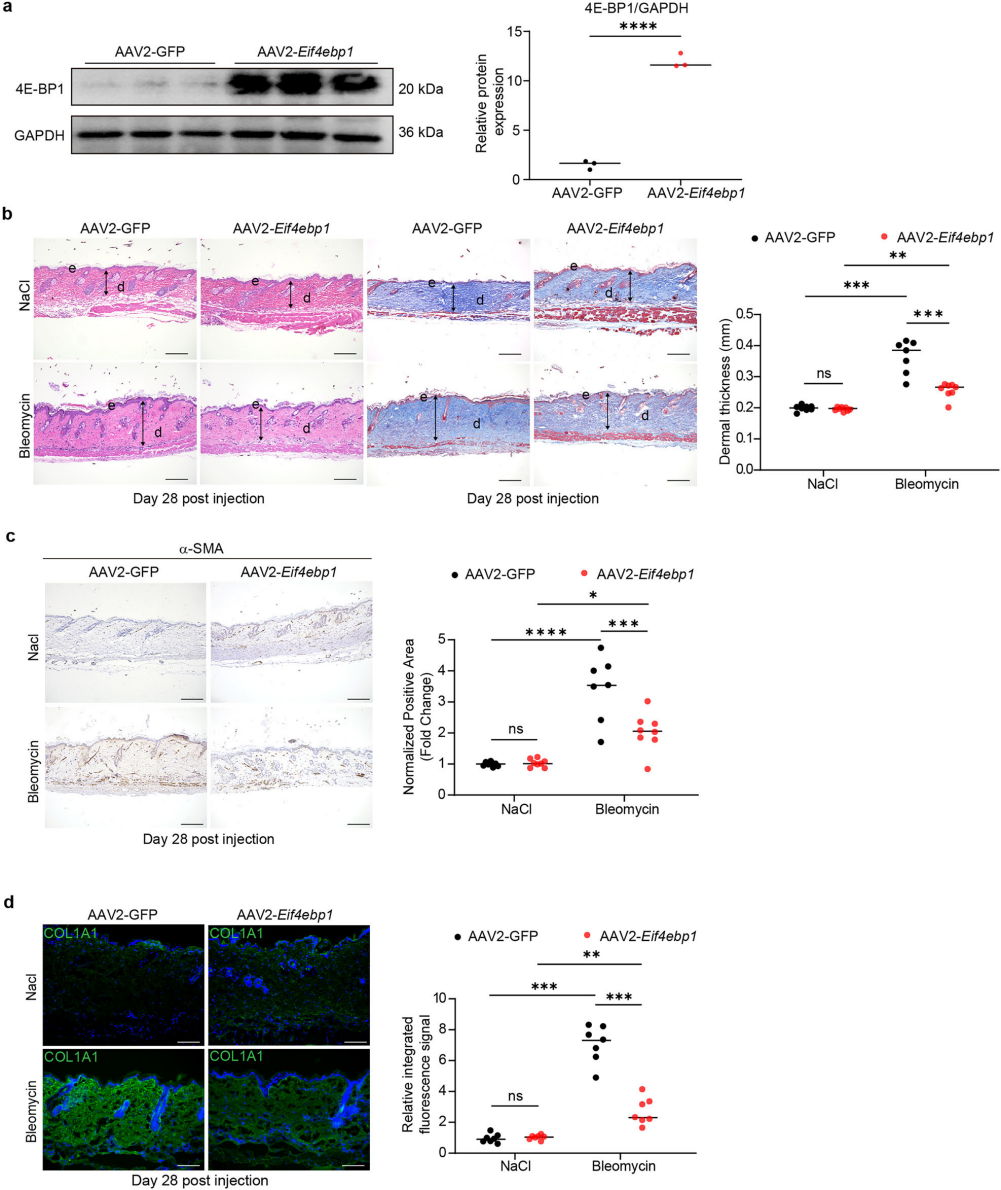
Overexpression of 4E-BP1 attenuates bleomycin-induced dermal fibrosis. **a** Western blot analysis of 4E-BP1 protein expression in skin tissues isolated from control and 4E-BP1 overexpression mice (*n* = 3). Quantification of 4E-BP1 expression. GAPDH was used as loading control. **b** Representative images of hematoxylin and eosin and Masson’s trichrome staining of dorsal skin sections from control and 4E-BP1 overexpression mice at day 28 post-injection of NaCl and bleomycin. Quantification of dermal thickness (*n* = 7–8). Scale bar, 250 μm. **c** Representative immunohistochemical staining of α-SMA in dorsal skin at day 28 post-injection (*n* = 7–8). Quantification of the percentage of α-SMA^+^ area. Scale bar, 250 μm. **d** Representative immunofluorescence staining for COL1A1 in dorsal skin at day 28 post-injection. Quantification of COL1A1 fluorescence intensity (*n* = 7). Scale bar, 500 μm. AAV adeno-associated virus, BP binding protein, d dermis, e epidermis, GFP green fluorescent protein, hpf high-power field, ns not significant, α-SMA α-smooth muscle actin. *P < 0.05, **P < 0.01, ***P < 0.001 by Student’s unpaired two-tailed *t* test (parts **a**–**d**).

We next asked whether enforced expression of 4E-BP1 could protect against dermal fibrosis. To address this, we used AAV-mediated gene delivery to induce sustained overexpression of 4E-BP1 in mouse dorsal skin (Fig. 7a), followed by daily intradermal bleomycin injections for 4 weeks to induce fibrosis (Supplementary Fig. 7a). Body weight monitoring revealed no significant differences between the AAV2-*Eif4ebp1* and AAV2-GFP groups (Supplementary Fig. 7a).

Histological and molecular analyses were performed to assess fibrotic remodeling. H&E staining of lesional skin revealed a marked reduction in dermal thickness in 4E-BP1-overexpressing animals compared with AAV2-GFP mice (Fig. 7b). These findings were corroborated by Masson’s trichrome staining, which showed significantly diminished deposition of collagen-rich ECM in 4E-BP1-overexpressing skin (Fig. 7b). The dermis appeared more loosely organized, and fibrotic lesions were less extensive and less cellular (Fig. 7b), indicating attenuated fibroblast activation and matrix accumulation.

We further assessed the expression of the myofibroblast marker α-SMA and COL1A1. Immunohistochemical staining and western blot analysis demonstrated a striking reduction in α-SMA-positive myofibroblasts in the dermis of 4E-BP1-overexpressing mice (Fig. 7c and Supplementary Fig. 7b), accompanied by lower COL1A1 expression levels (Fig. 7d). These data suggest that translational repression via 4E-BP1 overexpression limits fibroblast-to-myofibroblast transition and suppresses fibrotic ECM production.

### mTORC1/4E-BP1 signaling controls fibroblast collagen synthesis

**Fig. 8:**
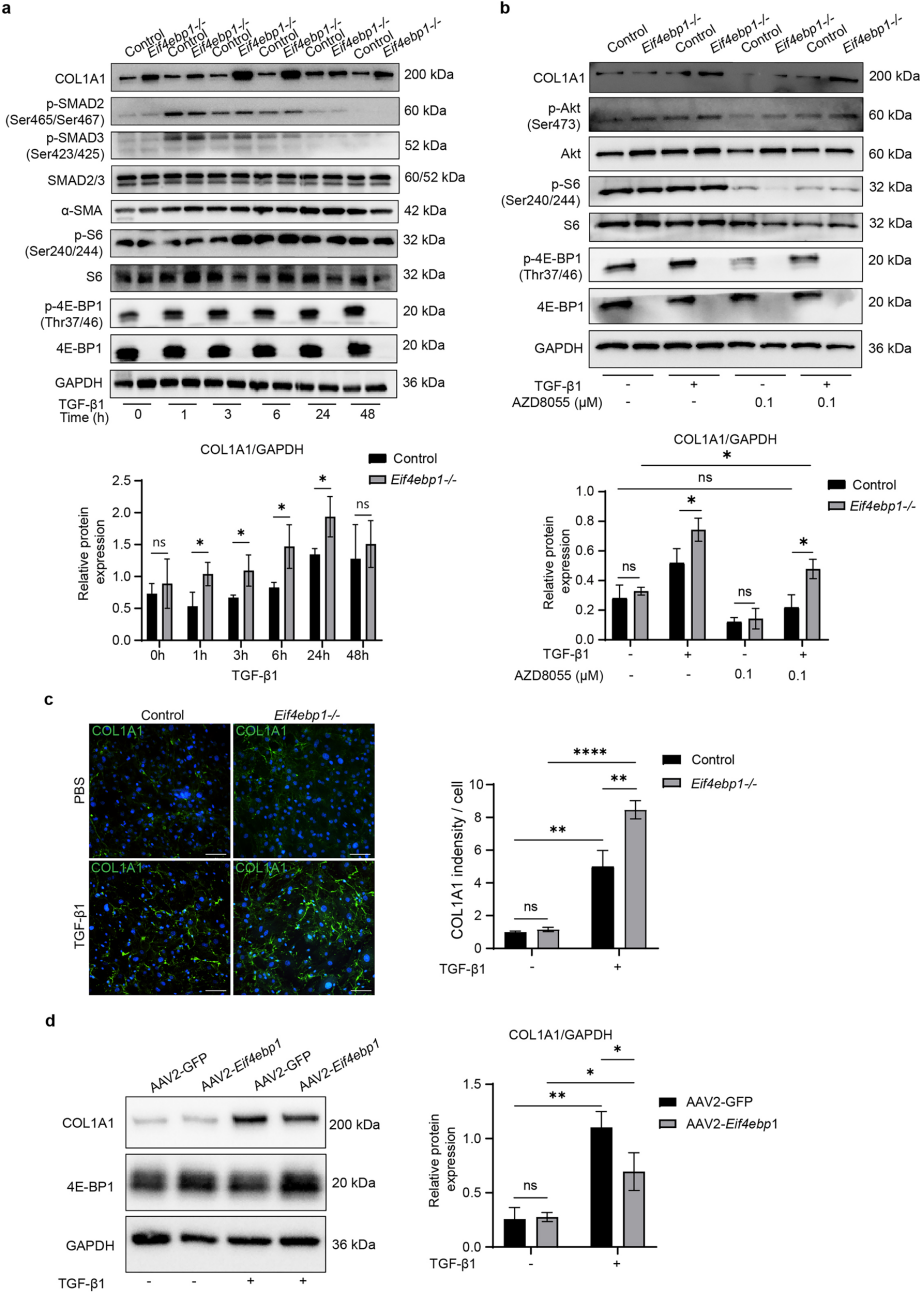
4E-BP1 knockout promotes type I collagen synthesis in fibroblasts. **a** Representative western blot analysis of phosphorylated SMAD2 (p-SMAD2 Ser465/Ser467), phosphorylated SMAD3 (p-SMAD3 Ser423/Ser425), α-smooth muscle actin (α-SMA), p-S6 Ser240/Ser244, and p-4E-BP1 Thr37/46 proteins. Primary skin fibroblasts isolated from control and *Eif4ebp1*^−^/^−^ mice were stimulated with transforming growth factor-β1 (TGF-β1) for indicated durations (*n* = 3). GAPDH was used as loading control. Statistical analysis of COL1A1 protein expression. **b** Western blot analysis of p-AKT, p-SMAD3, p-S6, and p-4EBP1 proteins. Fibroblasts were stimulated with TGF-β1 with or without AZD8055 pretreatment for 1 h (*n* = 3). Statistical analysis of COL1A1 protein expression. **c** Representative immunofluorescence staining of COL1A1 in fibroblasts after treatment with TGF-β1. Quantification of average COL1A1 fluorescence intensity (*n* = 3). Scale bar, 200 μm. **d** Western blot analysis of COL1A1 and 4E-BP1 proteins. Fibroblasts were infected with adeno-associated virus (AAV) to overexpress 4E-BP1 (*n* = 3). Statistical analysis of COL1A1 protein expression. BP binding protein, hpf high-power field, ns not significant. *P < 0.05, **P < 0.01, ***P < 0.001 by Student’s unpaired two-tailed *t* test (parts **a**-**d**).

To mechanistically link the observed in vivo fibrotic phenotype to translational control in fibroblasts, we conducted studies using primary dermal fibroblasts isolated from wild-type and 4E-BP1-deficient mice. Upon stimulation with TGF-β1, a central profibrotic cytokine, phosphorylation of the canonical downstream effector SMAD2/3 was induced, indicating intact TGF-β signaling. Interestingly, TGF-β1 stimulation also led to activation of mTOR signaling, as evidenced by increased phosphorylation of downstream effectors, including p-4E-BP1 and p-S6 (Fig. 8a and Supplementary Fig. 8a). In control fibroblasts, TGF-β1 promoted the expression of COL1A1 and α-SMA. Notably, *Eif4ebp1*⁻/⁻ fibroblasts displayed a modest, but not statistically significant, increase in baseline COL1A1 protein levels, which were markedly enhanced upon TGF-β1 treatment compared with wild-type cells (Fig. 8a,c and Supplementary Fig. 8b). By contrast, enforced overexpression of 4E-BP1 attenuated TGF-β1-induced COL1A1 expression (Fig. 8d). Collectively, these results suggest that loss of 4E-BP1 amplifies fibroblast responsiveness to profibrotic cues, providing direct evidence that 4E-BP1 level regulates collagen synthesis in skin fibroblasts.

To test whether these effects were mediated through mTORC1-dependent translational control, we treated primary dermal fibroblasts with the mTORC1 inhibitor AZD8055. Treatment with AZD8055 effectively suppressed mTOR activity, as evidenced by a marked reduction in p-S6 and p-4E-BP1. At high concentrations, AZD8055 significantly inhibited TGF-β1-induced COL1A1 expression (Fig. 8b), indicating that mTORC1 activity is required for full fibroblast activation and collagen production. Interestingly, at lower concentrations, COL1A1 expression remained elevated in *Eif4ebp1*^−^/^−^ fibroblasts, suggesting that the absence of 4E-BP1 derepresses mRNA translation and partially bypasses the inhibitory effects of mTORC1 blockade. Collectively, although the in vivo fibrotic phenotype reflects integrated multicellular responses, these in vitro findings demonstrate 4E-BP1 controls fibroblast activation and collagen synthesis, which is at least in part derepressing mTORC1-driven translational output.

### 4E-BP1-mediated fibrotic effects are conserved in human

**Fig. 9:**
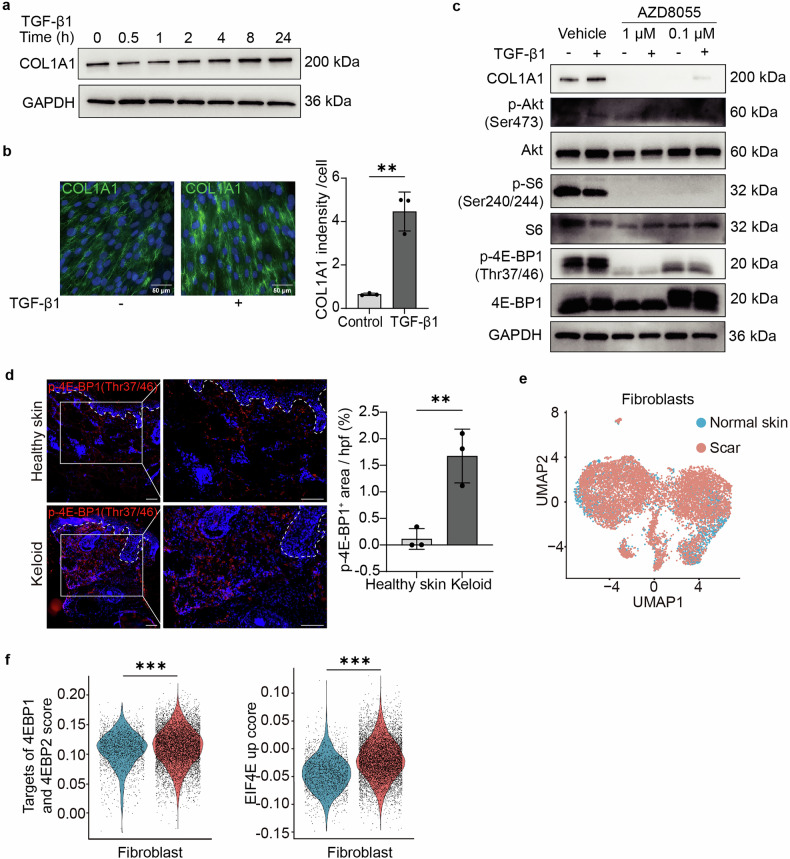
4E-BP1 regulates type I collagen expression in human fibroblasts and human fibrotic skin lesions. **a** Western blot analysis of COL1A1 expression in HDFs after stimulation with transforming growth factor-β1 (TGF-β1) for indicated durations. **b** Representative immunofluorescence staining of COL1A1 in HDFs after treatment with TGF-β1. Scale bar, 50 μm. Quantification of average COL1A1 fluorescence intensity (*n* = 3). **c** Western blot analysis of p-AKT, p-SMAD3, p-S6, p-4E-BP1 proteins. HDFs were stimulated with TGF-β1 with or without AZD8055 pretreatment (*n* = 3). **d** Representative immunofluorescence staining of p-4E-BP1 Thr37/46 in human healthy skin and keloid scar. Scale bar, 100 μm. Quantification of p-4E-BP1^+^ area per hpf (*n* = 3). **e** Uniform manifold approximation and projection (UMAP) of the fibroblasts in normal skin and scar. **f** Enrichment of 4E-BP1/4EBP2/eIF4E regulated gene signatures in human fibrotic skin lesions. BP binding protein, HDF human dermal fibroblast. *P < 0.05, **P < 0.01, ***P < 0.001 by Student’s unpaired two-tailed t test (parts **b**, **d** and **f**).

To investigate whether mTORC1 signaling contributes to fibrotic responses in human fibroblast cells, we stimulated primary HDFs with TGF-β1. Immunofluorescence staining showed increased phosphorylation of 4E-BP1 and S6, indicating robust mTORC1 activation upon TGF-β1 stimulation (Supplementary Fig. 8c). This led to a marked induction of COL1A1 expression (Fig. 9a,b), accompanied by a significant increase at the RNA level (Supplementary Fig. 8d). Co-treatment with the mTOR inhibitor AZD8055 effectively suppressed mTORC1 signaling, as evidenced by reduced phosphorylation of downstream effectors, and significantly decreased COL1A1 expression (Fig. 9c). These results indicate that mTORC1 activation is required for TGF-β1-induced fibrotic activation of HDFs.

To further assess the relevance of our findings in human fibrotic skin, we performed immunostaining for p-4E-BP1 in human keloid tissues. This analysis revealed markedly elevated p-4E-BP1 levels in keloid tissues (Fig. 9d), indicating activation of the pathway. We further analyzed single-cell transcriptomic data sets comparing normal skin and pathological scars, including hypertrophic scars and keloids^39^. Fibroblasts displayed similar overall distribution patterns across normal and scar tissues. However, gene signatures indicative of 4E-BP1/4E-BP2 deficiency and eIF4E overexpression, reflective of enhanced cap-dependent translation, were markedly enriched in scar-associated fibroblasts (Fig. 9e,f). Collectively, these data suggest persistent mTORC1 activation following skin injury and fibrotic progression. The enriched translation-related gene signatures in the scar microenvironment highlight the critical role of mTORC1-mediated 4E-BP1 inhibition in modulating wound healing and fibrotic remodeling in human skin.

## Discussion

mTORC1 integrates environmental and metabolic inputs to orchestrate cell growth, proliferation, and tissue repair^46^. Among its downstream effectors, 4E-BP1 acts as a translational repressor by inhibiting translation, thereby functioning as a critical checkpoint in mTORC1-regulated protein synthesis^12,47^. Here, we identify 4E-BP1 as a pivotal regulator of skin wound healing and fibrotic remodeling. We demonstrate that mTORC1 activation in the wound environment induces 4E-BP1 phosphorylation. Genetic ablation of 4E-BP1 mimics full mTORC1-mediated phosphorylation, leading to accelerated re-epithelialization, enhanced granulation tissue formation, and increased fibrotic matrix deposition. Overexpression of 4E-BP1 resulted in impaired wound healing. Single-cell transcriptomic analyses of human scar and keloid tissues enriched gene sets with 4E-BP1 loss or eIF4E overexpression, supporting the clinical relevance of our findings. Collectively, these results establish the mTORC1/4E-BP1/eIF4E signaling axis as a regulatory rheostat that balances effective tissue regeneration with the risk of fibrotic progression. Modulating 4E-BP1 activity may thus represent a promising therapeutic strategy to enhance wound repair while limiting pathological scarring.

Although systemic mTOR inhibition has been associated with lifespan extension^48,49^, accumulating evidence demonstrates that locally balanced mTORC1 activity is essential for optimal tissue repair. Genetic inactivation of mTOR leads to impaired healing response^16,21,50,51^. Although the specific contributions of mTORC1 downstream effectors to tissue repair remain elusive, ribosomal protein S6, another target of mTORC1, is phosphorylated in response to skin injury and marks a perilesional activation zone^19,41^. However, recent findings indicate that p-S6 functions as a modulator rather than a primary driver of the healing process^41^. Clinically, mTORC1 inhibitors such as rapamycin have been shown to impair wound healing^52,53^. However, rapamycin appears to inhibit 4E-BP1 phosphorylation partially^50^, the specific role of 4E-BP1 in skin repair has remained unresolved. Our study identifies 4E-BP1 as a pivotal molecular checkpoint that governs both wound repair and scar formation, revealing a previously unappreciated role for 4E-BP1 within the mTORC1 signaling cascade in coordinating skin reparative and fibrotic outcomes.

Our findings uncover a previously underappreciated role for 4E-BP1 in regulating wound angiogenesis. VEGF-A, a key pro-angiogenic factor, is a well-established translational target of the 4E-BP1/eIF4E axis^44^. We provided direct evidence that genetic ablation of 4E-BP1, which functionally mimics constitutive phosphorylation and release from eIF4E, results in elevated VEGF-A protein levels and enhanced neovascularization. Conversely, enforced overexpression of 4E-BP1 suppresses VEGF-A expression and reduces angiogenic activity in the wound bed. Notably, in diabetic wounds, in which impaired 4E-BP1 phosphorylation and reduced VEGF-A expression are observed, angiogenesis is also compromised, supporting the functional link between 4E-BP1-mediated translational control and vascular regeneration^27,29^. Together, these findings highlight the 4E-BP1/eIF4E axis as a critical regulator of angiogenic responses during tissue repair and suggest that dysregulation of this pathway may underlie defective vascularization in chronic wounds. Targeting 4E-BP1 activity could therefore represent a promising strategy to restore both epithelial integrity and vascular function in impaired healing conditions.

The composition and dynamics of ECM are pivotal determinants of wound resolution, scar formation, and fibrotic progression^54,55^. mTORC1 signaling has been implicated in regulating cell-cycle progression and ECM-associated gene expression, including fibronectin, collagen, and α-SMA^30,56,57^. However, the specific contributions of downstream effectors remain insufficiently defined. In our study, genetic ablation of 4E-BP1 resulted in disorganized collagen fibrillogenesis, characterized by heterogeneous fibril diameters and disrupted ultrastructure, underscoring a critical role for the mTORC1/4E-BP1 pathway in ECM remodeling during wound repair. Conversely, overexpression of 4E-BP1 suppressed collagen I production and reduced α-SMA expression, leading to impaired granulation tissue formation and delayed wound closure. These findings position 4E-BP1 as a key regulator linking mTORC1 signaling to ECM output. Notably, the mTORC1/4E-BP1 axis has been shown to modulate collagen synthesis by in vitro lung fibroblasts, suggesting a conserved role in fibrotic tissue remodeling^31^. Clinically, hypertrophic scars and keloids remain difficult to treat. Our observation that 4E-BP1 modulates fibroblast-derived collagen synthesis through mTORC1-dependent phosphorylation suggests a potentially targetable mechanism in pathological fibrosis and scarring. Fine-tuning 4E-BP1 activity or abundance may offer a strategy to rebalance ECM deposition, enhancing regenerative repair while minimizing fibrotic outcomes. Thus, the mTORC1/4E-BP1 axis emerges as a promising therapeutic target in both chronic wound care and fibroproliferative skin disorders.

We think that these divergent outcomes resulting from modulated 4E-BP1 expression are mechanistically linked to changes in the eIF4E/4E-BP1 ratio, a key determinant of cap-dependent translation. This ratio dictates the availability of eIF4E for initiating protein synthesis and thereby modulates the translational landscape during tissue repair. Supporting this concept is the finding in tumor study, which demonstrated that an elevated eIF4E/4E-BP1 ratio confers resistance to mTOR inhibition by sustaining translation of growth-promoting mRNAs^58,59^. In addition, 4E-BP1 or eIF4E loss confers whole-body metabolic alteration^42,60^. Analogously, our data indicate that a similar mechanism operates in the context of wound healing, in which a shift in this ratio influences wound cell proliferation, migration, ECM remodeling, and fibrotic response. Collectively, these findings position the mTORC1/4E-BP1/eIF4E axis as a translational rheostat that governs both the rate and quality of tissue regeneration.

Despite these functional insights, several limitations remain. The downstream translational targets through which 4E-BP1 modulates fibroblast fate decisions and collagen biosynthesis remain incompletely defined. In this study, proteomic analysis of wound tissues was performed. Although activation of fibrotic signaling pathways was observed in 4E-BP1-deficient tissues, direct translational targets of 4E-BP1 could not be identified, largely due to methodological constraints of the current experimental setting. Future studies using translatome profiling approaches such as ribosome foot-printing may help identify these effectors^61^. Second, the specific roles of 4E-BP1 in distinct cellular subtypes within the wound environment require further elucidation. Moreover, although genetic approaches provide proof-of-concept, the clinical translation of these findings requires development of pharmacological agents that selectively modulate 4E-BP1 activity^62^. Such selective modulators may allow for precise control of wound healing dynamics, offering a strategy to enhance regeneration while minimizing the risk of fibrosis or keloid formation.

In summary, we identify 4E-BP1 as a key modulator of skin wound healing and fibrosis, linking mTORC1 signaling to translational control of regenerative and fibrotic processes. Selective modulation of 4E-BP1 activity may therefore offer a powerful tool to fine-tune the reparative process and limit fibrotic sequelae. Indeed, therapeutic strategies targeting pan-PI3K/mTOR, the upper regulator of 4E-BP1, are already being explored in clinical trials for idiopathic pulmonary fibrosis^63^, underscoring their translational potential. Future studies should aim to elucidate the spatiotemporal dynamics of 4E-BP1 activity in wound healing and define the precise translational programs it governs across different cellular compartments, thereby harnessing the therapeutic promise of this pathway for clinical wound management.

## Supplementary information

Supplementary Information
