# Supplementary material for: 4E-BP1 acts as a molecular rheostat balancing regenerative healing and fibrotic scarring
Source: Exp Mol Med. 2026 Jun 2;58(6):1772–88. doi: 10.1038/s12276-026-01724-0 (PMC13324001; doi:10.1038/s12276-026-01724-0)
Supplement: Supplementary file 1 — Supplementary Information [file 12276_2026_1724_MOESM1_ESM.pdf]

4E-BP1 acts as a molecular rheostat balancing regenerative healing and fibrotic scarring

SUPPLEMENTARY MATERIALS AND METHODS

Proteomic analysis

Skin wounds harvested at 7 day post-injury were subjected to protein extraction. Following quantitative determination, proteins were denatured and reduced using urea and dithiothreitol, then alkylated with iodoacetamide. Subsequently, the proteins were digested with trypsin for 12 hours, and the resulting peptides were desalted. LC-MS/MS analysis was performed utilizing an EASY-nanoLC 1200 system (Thermo Fisher Scientific) coupled to an Exploris 480 mass spectrometer (Thermo Fisher Scientific) for Data-Independent Acquisition (DIA). Separation was achieved using an Acclaim PepMap RSLC nano Viper analytical column (75 µm × 25 cm). Raw data files were processed using Spectronaut software for database searching and protein/peptide quantification. MS/MS spectra were searched against the UniProtKB/Swiss-Prot human database. GO (http://geneontology.org)enrichment analysis, KEGG pathway enrichment (https://www.kegg.jp/kegg/mapper.html), and STRING protein interaction (STRING: functional protein association networks) analysis were conducted.

Polysome profiling

Polysome profiling was performed to assess global translational activity. Wound tissues at day 7 post-injury were treated with cycloheximide (100 µg/mL) to stabilize ribosome–mRNA complexes, harvested, snap-frozen, and lysed on ice in polysome lysis buffer containing HEPES, KCl, $MgCl_2$, DTT, Triton X-100, and cycloheximide. Lysates were clarified by centrifugation at 4 °C, and RNA content was normalized based on absorbance at 260 nm. Equal amounts of lysate were loaded onto linear sucrose gradients and subjected to ultracentrifugation at 4 °C. Gradients were fractionated using a peristaltic pump while monitoring absorbance at 254 nm to generate polysome profiles.

Lentiviral production and generation of *EIF4EBP1*-knockout human keratinocyte cell Line

Single guide RNAs (sgRNAs) targeting the human EIF4EBP1 gene were designed using the CRISPRDB web server (https://crisprdb.org) and cloned into the lentiCRISPR v2 plasmid. The lentiCRISPR v2 vector was a gift from Feng Zhang (Addgene plasmid #52961; RRID: Addgene_52961). Lentiviral particles were produced by co-transfecting HEK293T cells with 1.5 μg lentiCRISPR v2-sgEIF4EBP1, 0.9 μg psPAX2 packaging plasmid, and 0.6 μg pMD2.G envelope plasmid using Lipo8000 transfection reagent (Beyotime, C0533). Viral supernatants were collected 48-72 h after transfection.

The following sgRNAs targeting EIF4EBP1 were used: sgRNA-1: 5′-GACTACAGCACGACCCCCGG-3′; sgRNA-2: 5′-GGGATCTGCCCACCATTCCG-3′. An empty lentiCRISPR v2 vector was used to generate control cell lines. HaCat human keratinocyte cells were transduced and selected with puromycin (2 μg/ml) for 48-72 h, followed by single-cell cloning by limiting dilution in 96-well plates. Successful knockout of EIF4EBP1 was confirmed by immunoblotting and functional assays.

Supplementary Table 1. Gene set information from GSEA.

Gene set name	Description	Genes
**EIF4E_UP**	Genes up-regulated in HMEC cells (primary mammary epithelium) upon over-expression of EIF4E	ACYP1, PHYKPL, AHSA1, ANKRD16, ATP5MF, ATP8B3, BMP3, BOLA1, SNRNP25, PAGR1, NTPCR, RIPOR3, TMEM230, DNPH1, C8orf33, CACNB3, CAMK2N2, CARTPT, CFHR1, CHCHD1, CKMT2, CNTN4, EIF4A3, DEDD, DNAJB5, DXO, FABP7, FAM117A, FBXL13, FBXO4, FETUB, FKBP1B, MIRLET7BHG, FN3KRP, FOXD2, GEMIN6, GTF2H5, NOP16, IDH1, IL18, ISOC2, ITPK1, JOSD1, KCNC2, KLF8, LAMC3, UQCC4, C6orf226, PNO1, DPY30, LST1, MAP2K3, MAPK8IP1, MDH2, METTL1, STPG2, CCDC187, MON1A, MRPL17, MRPL30, MRPS35, MS4A1, MTX2, MYH6, NDRG1, NEGR1, NKX2-8, NPM3, NUDCD2, OSBPL10, PFDN2, PMM2, PPCS, PRSS1, PTS, RGS3, RSAD1, SLC25A30, SNTB1, SNX8, SRXN1, ZNRD2, BHLHE40, TAF11, TALDO1, TAX1BP3, TMEM11, TP53I11, TST, TUFM, UBL4A, UBFD1, UCHL3, HAUS7, UROS, VIPR2, VPS28, VPS72, ZBED2, ZNF576

**COLINA_TARGETS_OF_4EBP1_AND_4EBP2**	Genes up-regulated in MEF cells (embryonic fibroblast) with double knockout of the translation repressors 4EBP-1 and 4EBP-2	PAFAH2, PPP1R8, SMPDL3B, RAB28, GRWD1, PHKB, XPO1, HOXB2, , ESPL1, KAZALD1, HAT1, CDC20, SLCO3A1, FOXP1, ATAD3A, NUSAP1, RCC2, ZZZ3, KIF22, PHKA2, ALDH7A1, PWP2, ACADM, FOXP2, ADA, ADAMTS1, GLA, ALDH2, ANG, FABP4, BIRC5, ATM, ATP1B1, AUP1, B2M, BAG1, BCAT1, BID, FABP7, BMI1, C3, C4B, C4BPA, CCNA2, CCND1, CD14, SCARB2, CD59, CDC25B, CDH11, CDO1, TPP1, COL2A1, CP, CR1L, CRYGS, CRYZ, CTSH, DBP, ECT2, EIF1AX, EMB, ENO2, ENO3, NR2F1, KHDRBS3, CELF2, EYA1, F3, SESN1, FGF18, FGF7, FOXC2, FOXM1, FST, GALT, GBP2, GBP2, PDPN, GPAA1, GRK5, GPX3, GRN, HLA-B, HLA-A, HLA-E, HELLS, HEXB, FOXD1, HMMR, FOXA1, HOXA2, HOXA5, ID2, ID3, ID4, IRGM, , IFIT2, IFNGR1, IGFBP2, IRGM, IL13RA1, IL1RN, IMPACT, IRX3, IRF9, ITIH2, SSPN, KRT10, KRT32, LAMP2, LBP, LCN2, CNMD, LGALS9, PSMB9, PSMB8, LTBP2, LXN, LY6E, GLCCI1, LY96, TM4SF1, MFAP2, MELK, FOXC1, MME, MMP3, MT1A, MT2A, NDN, NEK2, NEO1, NFIB, NFKB1, NQO2, NNMT, NPR3, NSG1, FXYD5, TNFRSF11B, SIGMAR1, P2RX4, PAM, PAX8, PDE1A, PFKL, PFN2, ABCB1, PRKCA, PLK1, PLSCR1, PRRX1, PNP, POLA1, POLE, PPFIBP2, LGALS3BP, TMEM47, PTPRF, KIF20A, RAD1, RAD51, RAD9A, RBMX, MRPL23, SUB1, TRIM5, S100A1, S100A13, CCL5, CXCL6, SELENOW, SFRP4, SLC12A2, SNRPD1, SNRPE, SOD3, TOR1AIP1, STAT1, STC2, AURKB, BHLHE40, SDC4, MAPRE2, TACC3, TBX1, PHLDA1, TRIM25, TFPI, ZNF322, TGM2, THBD, THBS1, DLGAP5, SCARA3, TOP2A, TTK, VAMP3, VCAM1, VEGFB, FMNL3, CCN5, WNT5A, WNT5B, THOC1, HAUS1, PTGR3, ZNF35, LYPLAL1, ORMDL1, KYAT3, TUT4, ATP10D, BLVRB, PARP3, TCEAL1, ADGRG2, BACE1, ABCC4, RSPO2, OASL2P, OSR1, USP18, PARP12, ABCG2, , CDK8, PLOD2, CCNB1, CENPH, RACGAP1, ISLR, PON3, NIT1, TCIRG1, DBF4, PRMT5, TIMM10, TOR3A, GALM, PON2, SLC41A2, ACSL5, CREG1, POSTN, C1S, C1R, SRPX, PBK, CAMK1, DHRS1, PNRC2, PPP1R3C, ISG15, RECK, VAMP5, NUDT5, RFX5, EGFL6, SKAP2, RFK, IRGM, RAMP2, GBP4, IFT20, LGALS8, UBQLN1, MRPL37,

		DIAPH3, TSPAN6, PALS2, RIPK3, MGST1, GMNN, CDC42SE1, ZBP1, TRIM54, RNASE4, NAMPT, CENPK, , SLC29A1, PERP, SVEP1, DTD1, GNG11, NAT8B, IFITM3, DNAJC15, PYCR3, CD302, AIG1, RNF113A, APTX, EXOC2, AGGF1, NACC1, MFSD1, RNF128, ASF1B, SHISA5, NUF2, NDC80, MMACHC, FBXO5, LRRC40, SMARCA2, LPAR6, CDT1, UBE2T, PFDN1, GPX7, ARMCX2, DNAJC19, RTP4, ILF2, PLPP3, UPF3B, NRN1, HDDC3, ANLN, ABHD11, TMEM53, TMEM97, DDAH1, POLR2D, BST2, EXOSC8, PIR, TRIM32, SASH1, IFI35, GPRASP3, ASNSD1, FUCA1, OSBPL3, CA13, TPX2, PGM1, MTREX, HSDL2, PIP4P2, PCBD2, NDC1, KIF2C, VIT, TSPAN17, CLTB, HNRNPR, PHPT1, RBM12, SESN3, RAP2A, IFI27L2, ARFGAP2, CPT1C, SLC16A3, AKAP12, BICC1, SNURF, CHRAC1, TINAGL1, IFI44
**HALLMARK_MTORC1_SIGNALING**	Genes up-regulated through activation of mTORC1 complex	ABCF2, ACACA, ACLY, ACSL3, ACTR2, ACTR3, ADD3, ADIPOR2, AK4, ALDOA, ARPC5L, ASNS, ATP2A2, ATP5MC1, ATP6V1D, AURKA, BCAT1, BHLHE40, BTG2, BUB1, CACYBP, CALR, CANX, CCNF, CCNG1, CCT6A, CD9, CDC25A, CDKN1A, CFP, COPS5, CORO1A, CTH, CTSC, CXCR4, CYB5B, CYP51A1, DAPP1, DDIT3, DDIT4, DDX39A, DHCR24, DHCR7, DHFR, EBP, EDEM1, EEF1E1, EGLN3, EIF2S2, ELOVL5, ELOVL6, ENO1, EPRS1, ERO1A, ETF1, FADS1, FADS2, NIBAN1, FDXR, FGL2, FKBP2, G6PD, GAPDH, GBE1, GCLC, GGA2, GLA, GLRX, GMPS, GOT1, GPI, GSK3B, GSR, GTF2H1, HK2, HMBS, HMGCR, HMGCS1, HPRT1, HSP90B1, HSPA4, HSPA5, HSPA9, HSPD1, HSPE1, IDH1, IDI1, IFI30, IFRD1, IGFBP5, IMMT, INSIG1, ITGB2, LDHA, LDLR, LGMN, LTA4H, M6PR, MAP2K3, MCM2, MCM4, ME1, MLLT11, MTHFD2, MTHFD2L, NAMPT, NFIL3, NFKBIB, NFYC, NMT1, NUFIP1, NUP205, NUPR1, P4HA1, PDAP1, PDK1, PFKL, PGK1, PGM1, PHGDH, PIK3R3, PITPNB, PLK1, PLOD2, PNO1, PNP, POLR3G, PPA1, PPIA, PPP1R15A, PRDX1, PSAT1, PSMA3, PSMA4, PSMB5, PSMC2, PSMC4, PSMC6, PSMD12, PSMD13, PSMD14, PSME3, PSMG1, PSPH, QDPR, RAB1A, RDH11, RIT1, RPA1, RPN1, RRM2, RRP9, SC5D, SCD, SDF2L1, SEC11A, SERP1, SERPINH1, SHMT2,

		SKAP2, SLA, SLC1A4, SLC1A5, SLC2A1, SLC2A3, SLC37A4, SLC6A6, SLC7A11, SLC7A5, NHERF1, SORD, SQLE, SQSTM1, SRD5A1, SSR1, STARD4, STC1, STIP1, SYTL2, TBK1, TCEA1, TES, TFRC, TM7SF2, TMEM97, TOMM40, TPI1, TRIB3, TUBA4A, TUBG1, TXNRD1, UBE2D3, UCHL5, UFM1, UNG, USO1, VLDLR, WARS1, XBP1, YKT6
**HALLMARK_PI3K_AKT_MTOR_SIGNALING**	Genes up-regulated by activation of the PI3K/AKT/mTOR pathway	ACACA, ACTR2, ACTR3, ADCY2, GRK2, AKT1, AKT1S1, AP2M1, ARF1, ARHGDIA, ARPC3, ATF1, CAB39, CAB39L, CALR, CAMK4, CDK1, CDK2, CDK4, CDKN1A, CDKN1B, CFL1, CLTC, CSNK2B, CXCR4, DAPP1, DDIT3, DUSP3, E2F1, ECSIT, EGFR, EIF4E, FASLG, FGF17, FGF22, FGF6, GNA14, GNGT1, GRB2, GSK3B, HRAS, HSP90B1, IL2RG, IL4, IRAK4, ITPR2, LCK, MAP2K3, MAP2K6, MAP3K7, MAPK1, MAPK10, MAPK8, MAPK9, MAPKAP1, MKNK1, MKNK2, MYD88, NCK1, NFKBIB, NGF, NOD1, PAK4, PDK1, PFN1, PIK3R3, PIKFYVE, PIN1, PITX2, PLA2G12A, PLCB1, PLCG1, PPP1CA, PPP2R1B, PRKAA2, PRKAG1, PRKAR2A, PRKCB, PTEN, PTPN11, RAC1, RAF1, RALB, RIPK1, RIT1, RPS6KA1, RPS6KA3, RPTOR, SFN, SLA, SLC2A1, SMAD2, SQSTM1, STAT2, TBK1, THEM4, TIAM1, TNFRSF1A, TRAF2, TRIB3, TSC2, UBE2D3, UBE2N, VAV3, YWHAB

SUPPLEMENTARY FIGURE AND FIGURE LEGENDS

Supplementary Fig. 1

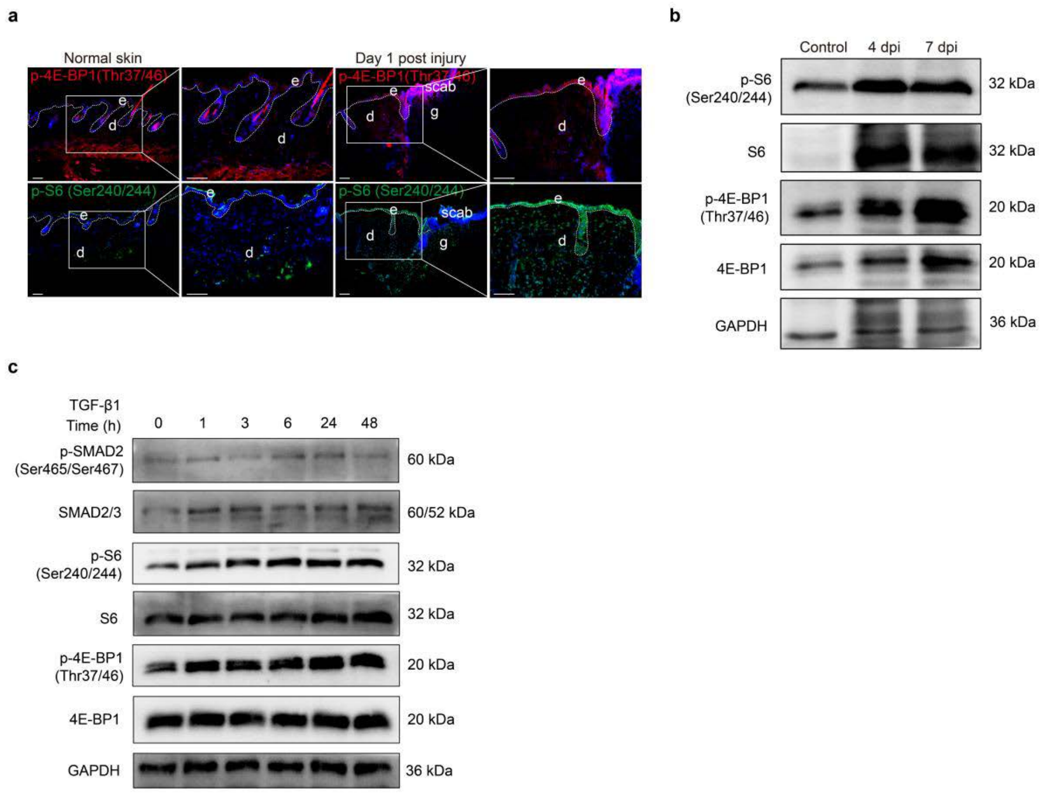

Supplementary Fig. 1 Western blot analysis of mTOR signaling activation in wound tissues and primary dermal fibroblasts. **a** Representative immunofluorescence staining of p-4E-BP1 Thr37/46 and p-S6 Ser240/244 in full-thickness skin wounds at day 1 dpi. Scale bar, 100 μm. **b** Western blot analysis of mTOR pathway-related protein expression in wound tissues. **c** Western blot analysis of mTOR pathway-related protein expression in primary mouse dermal fibroblasts treated with TGF-β1 (1 ng/mL).

Supplementary Fig. 2

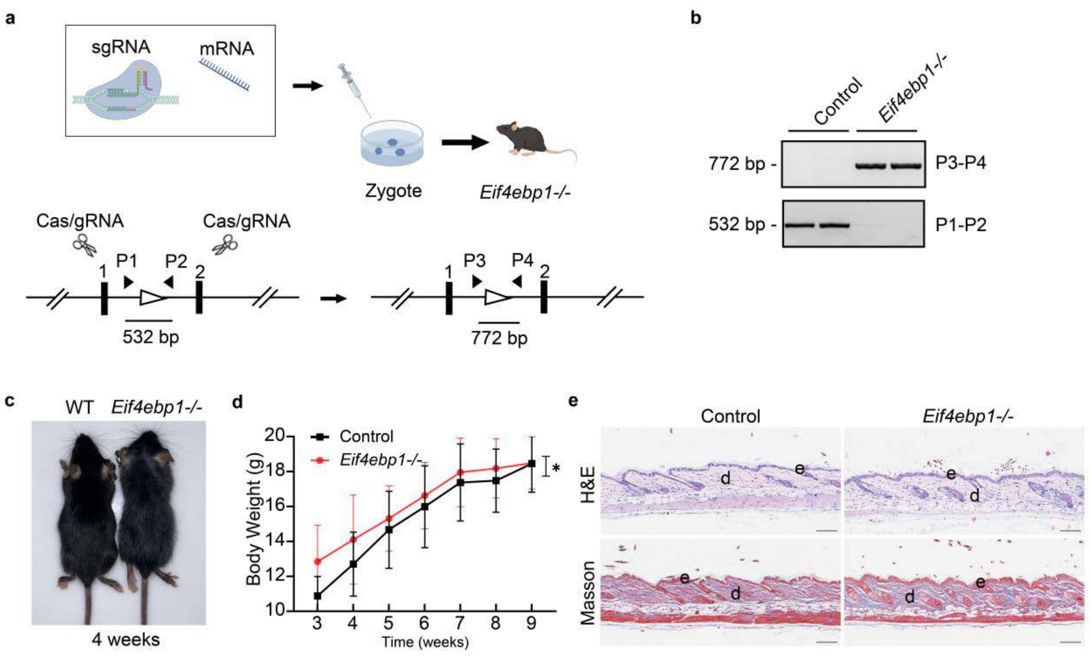

Supplementary Fig. 2 Establishment of *Eif4ebp1*$^{-/-}$ mice. **a** Schematic diagram illustrating the establishment *Eif4ebp1*$^{-/-}$ mice through CRISPR-Cas technology. **b** PCR analysis of *Eif4ebp1*$^{-/-}$ mice genotyping. **c** Images of wild-type (WT) and *Eif4ebp1*$^{-/-}$ mice. **d** Body weight changes in mice from weeks 3 to 8 (n = 7-8). **e** Representative H&E and Masson's staining of skin sections. Scale bar, 100 μm. d, dermis; e, epidermis. *p < 0.05, **p < 0.01, ***p < 0.001 by two-way ANOVA (**d**).

Supplementary Fig. 3

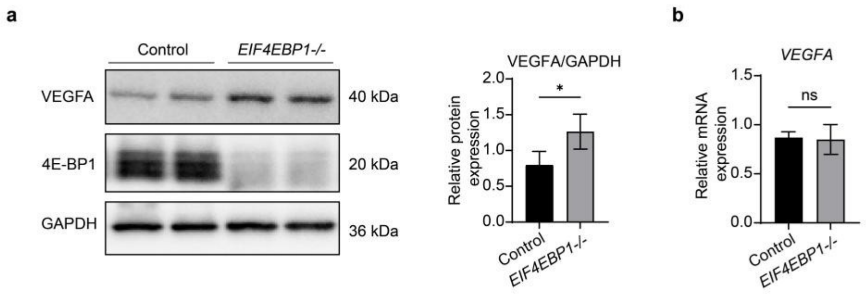

Supplementary Fig. 3 4E-BP1 knockdown increases VEGFA protein expression in HaCaT cells. **a** Western blot analysis of VEGFA expression following *EIF4EBP1* knockdown in HaCaT cells (n = 3). **b** RT-PCR analysis of *VEGFA* mRNA expression (n = 6). ns, not significant. *p < 0.05, **p < 0.01, ***p < 0.001 by Student's unpaired two-tailed t test.

Supplementary Fig. 4

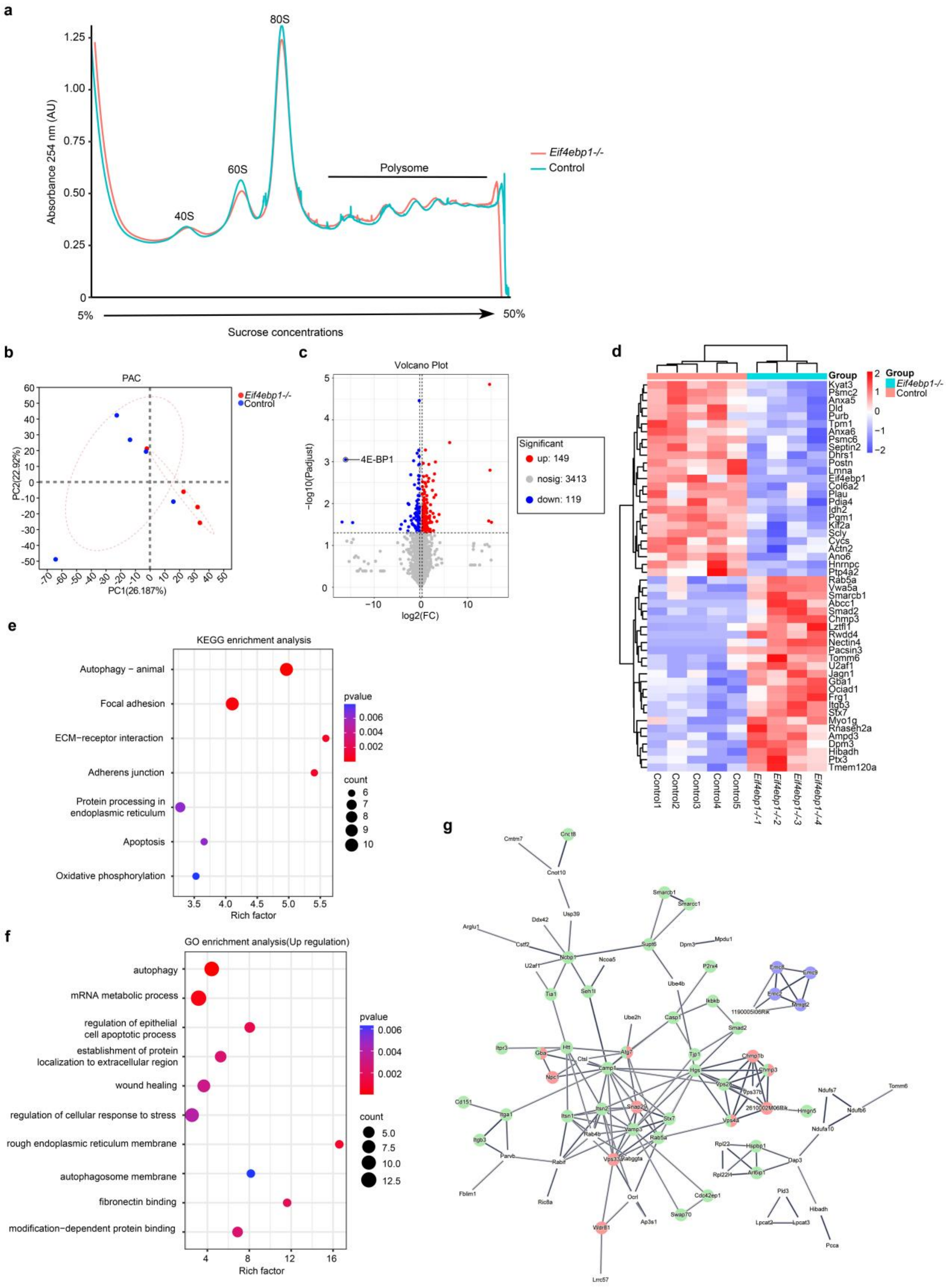

Supplementary Fig. 4 Polysome profiling and proteomic analysis of skin wounds in control and *Eif4ebp1*$^{-/-}$ mice at 7 dpi. **a** Representative polysome profiling analysis of global mRNA translation in mouse wounds from control and *Eif4ebp1*$^{-/-}$ mice at 7 dpi.

b Principal component analysis (PCA) was performed based on differentially expressed proteins in the wound tissues of the two groups. **c** Volcano plot of differentially expressed proteins. **d** Heatmap of significantly altered protein expression profiles (n = 5). **e** KEGG pathway enrichment analysis of key pathways modulated by *Eif4ebp1*$^{-/-}$. **f** GO enrichment analysis of the upregulated proteins in mouse wounds from control and *Eif4ebp1*$^{-/-}$ mice at 7 dpi. n=3 or 4. **g** STRING protein–protein interaction network of upregulated proteins (fold change >2) in *Eif4ebp1*$^{-/-}$ wounds, highlighting proteins involved in positive regulation of cellular processes (green), autophagy (red), and extracellular matrix organization (purple).

Supplementary Fig. 5

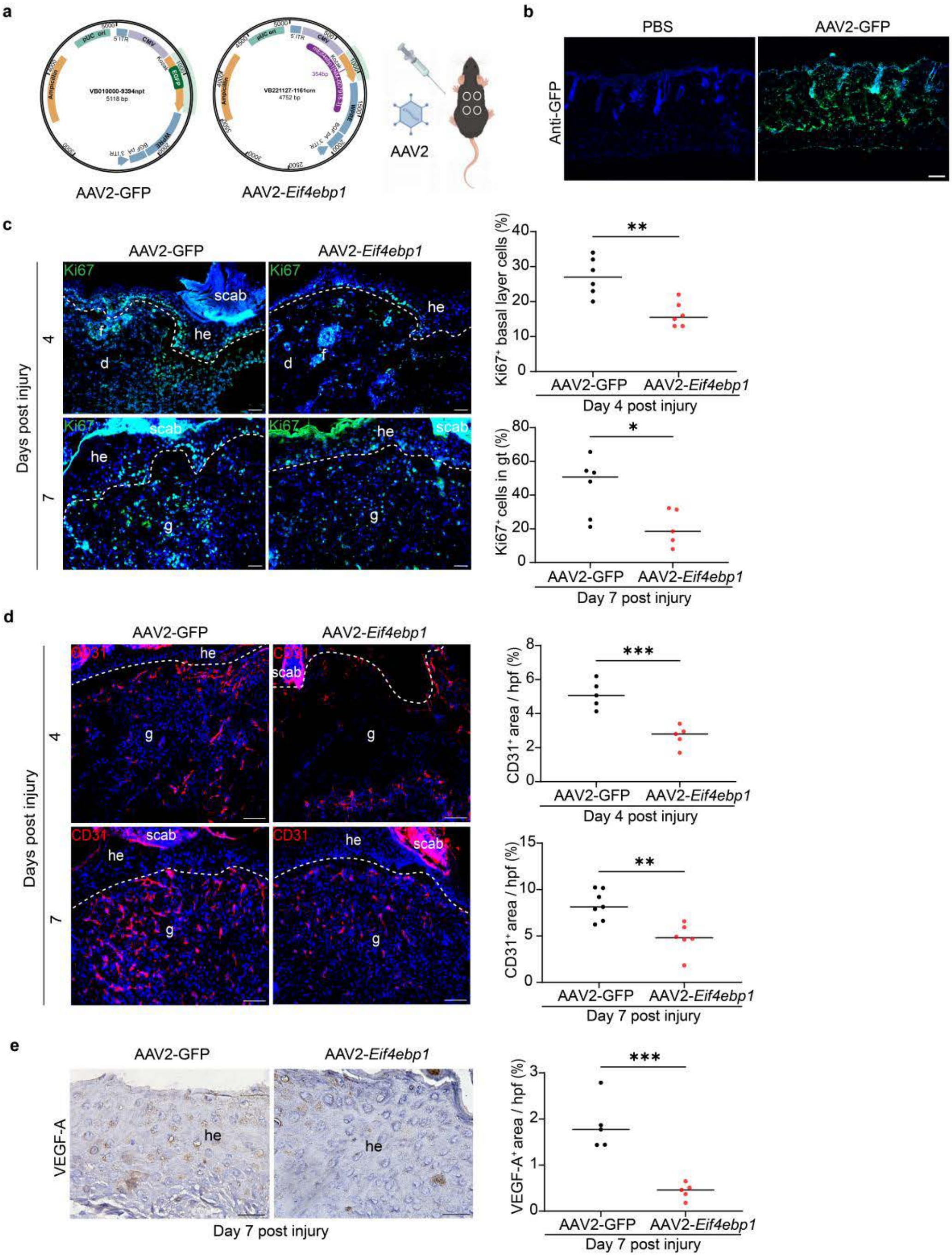

Supplementary Fig. 5 4E-BP1 overexpression inhibits cell proliferation and angiogenesis during wound healing. **a** Schematic of viral vector design and *in vivo* delivery. **b** Representative immunofluorescence images of GFP (anti-GFP) in uninjured mouse skin 2 weeks after intradermal injection of PBS or AAV2-GFP virus. Scale bar, 100 μm. **c** Representative immunofluorescence staining of Ki67 on wound sections at

4 and 7 dpi. Scale bar, 50 μm. Quantification of Ki67$^+$ cells (n = 5-6 wounds from 5 mice per group). **d** Representative immunofluorescence staining of CD31 on wound sections at 4 and 7 dpi. Scale bar, 100 μm. Quantification of CD31$^+$ area per hpf (n = 5-7 wounds from 5 mice per group). **e** Representative immunohistochemical staining of VEGF-A in he from wound sections at 7 dpi. Scale bar, 50 μm. Quantification of VEGF-A$^+$ area per hpf (n = 5 wounds from 5 mice per group). d, dermis; e, epidermis; g, granulation tissue; he, hyperproliferative epithelium. hpf, high power field. *p < 0.05, **p < 0.01, ***p < 0.001 by Student's unpaired two-tailed t test (**c-e**).

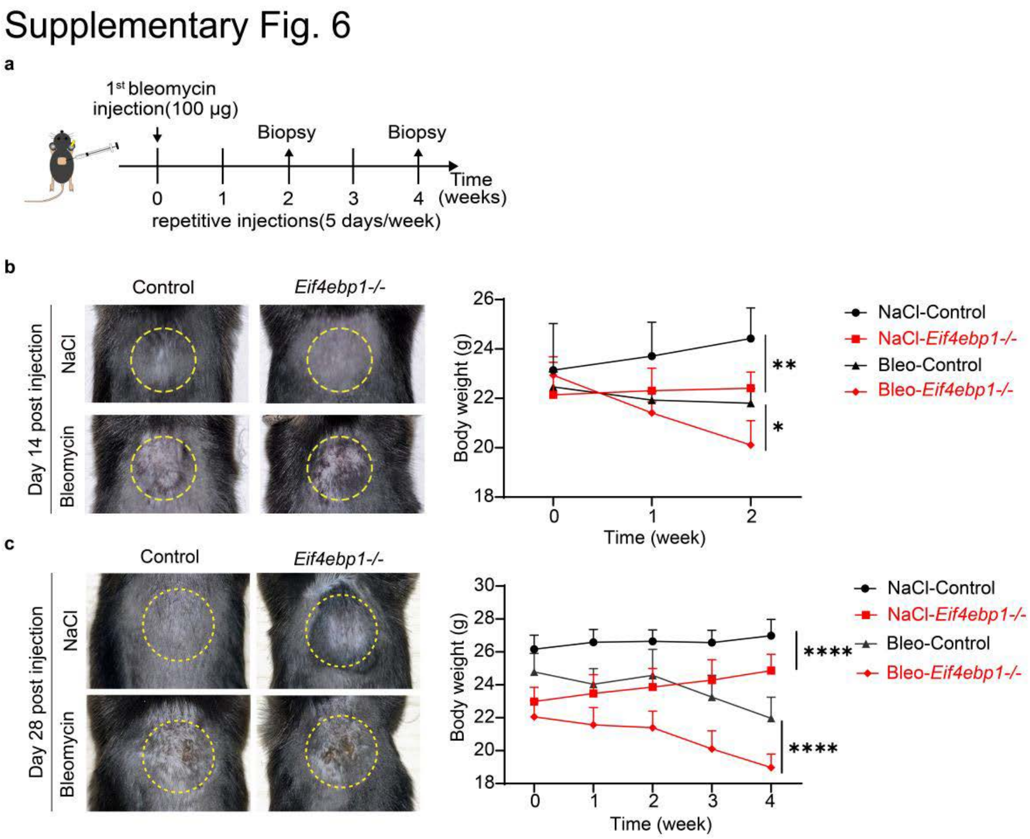

Supplementary Fig. 6 Analysis of bleomycin-induced dermal fibrosis in *Eif4ebp1*$^{-/-}$ mice**. a** Schematic diagram illustrating bleomycin-induced skin fibrosis model in mice. **b, c** Photographs of dorsal skin and body weight of *Eif4ebp1*$^{-/-}$ and control mice during bleomycin injection from weeks 2 to 4 (n = 3-8). GAPDH serves as loading control. *p < 0.05, **p < 0.01, ***p < 0.001 by two-way ANOVA (**b** and **c**).

Supplementary Fig. 7

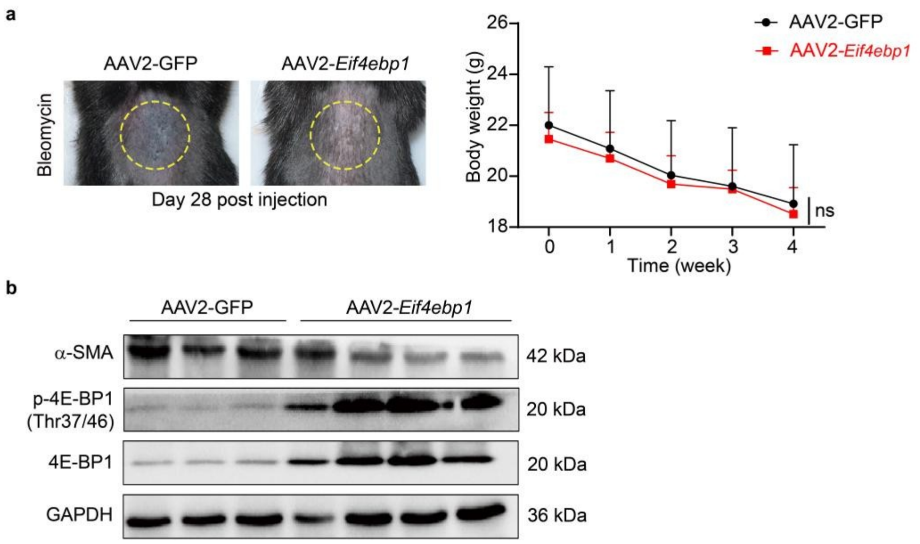

Supplementary Fig. 7 Analysis of bleomycin-induced dermal fibrosis in 4E-BP1 overexpressed mice. **a** Photographs of dorsal skin and body weight of 4E-BP1-overexpression and control mice at 4 weeks post-bleomycin injection (n = 7-8). **b** Western blot analysis of α-SMA expression in the dermis of 4E-BP1 overexpressed mice. GAPDH serves as loading control. *$p < 0.05$, **$p < 0.01$, ***$p < 0.001$ by two-way ANOVA (**a**).

Supplementary Fig. 8

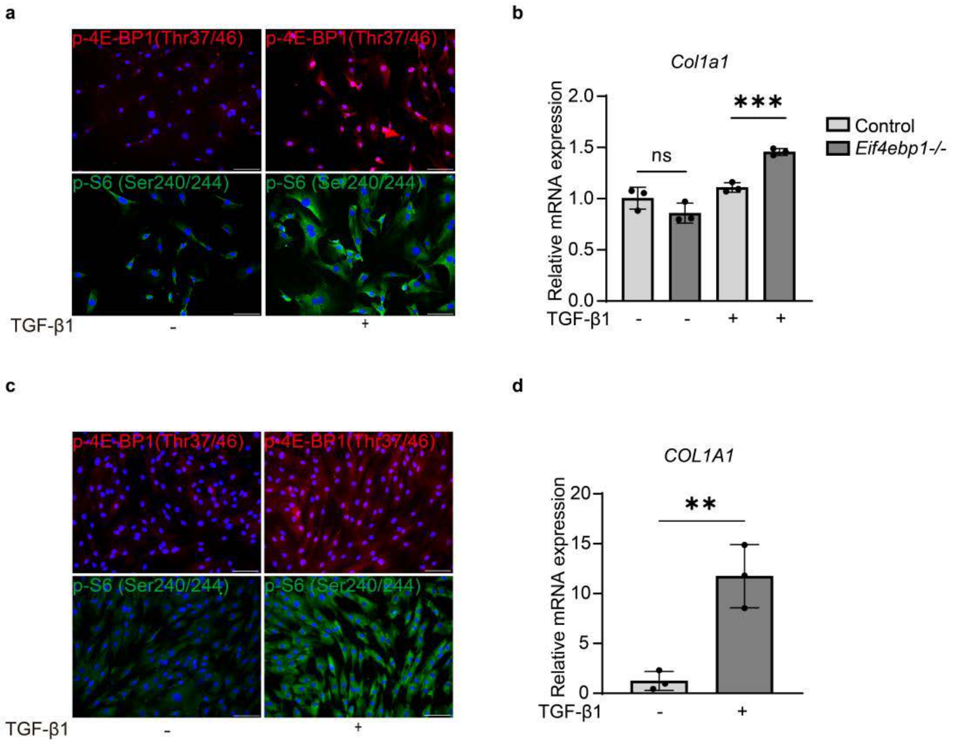

Supplementary Fig. 8 Analysis of collagen expression in primary dermal fibroblasts. **a** Immunofluorescence staining of p-4E-BP1 Thr37/46 and p-S6 Ser240/244 in primary mouse dermal fibroblasts. **b** qRT-PCR analysis of *Col1a1* mRNA levels in fibroblasts from *Eif4ebp1*$^{-/-}$ and wild-type controls with or without TGF-β1 treatment (n = 3). **c** Immunofluorescence staining of p-4E-BP1 Thr37/46p-S6 Ser240/244 in HDFs. **d** qRT-PCR analysis of *COL1a1* mRNA levels in HDFs with or without TGF-β1 treatment (n = 3). Scale bar, 100 μm. HDFs, human dermal fibroblasts; *p < 0.05, **p < 0.01, ***p < 0.001 by Student's unpaired two-tailed t test (**b** and **d**).
